# Major excursions in sulfur isotopes linked to permafrost change in Eurasia during the last 50,000 years

**DOI:** 10.1038/s41561-025-01760-x

**Published:** 2025-08-01

**Authors:** Rhiannon E. Stevens, Hazel Reade, Kerry L. Sayle, Jennifer A. Tripp, Delphine Frémondeau, Adrian Lister, Ian Barnes, Mietje Germonpré, Martin Street, Julian B. Murton, Simon H. Bottrell, Daniel H. James, Thomas F. G. Higham

**Affiliations:** 1 UCL Institute of Archaeology, London, UK; 2 Scottish Universities Environmental Research Centre, Rankine Avenue, East Kilbride, UK; 3 Department of Chemistry, University of San Francisco, San Francisco, CA USA; 4 Department of Earth Sciences, Natural History Museum, London, UK; 5 Royal Belgian Institute of Natural Sciences, Brussels, Belgium; 6 MONREPOS Archaeological Research Center and Museum for Human Behavioural Evolution, Römisch-Germanisches Zentralmuseum, Leibniz-Research Institute for Archaeology, Neuwied, Germany; 7 Department of Geography, University of Sussex, Brighton, UK; 8 School of Earth and Environment, University of Leeds, Leeds, UK; 9 Department of Evolutionary Anthropology, Faculty of Life Sciences, University of Vienna, Vienna, Austria; 10 Human Evolution and Archaeological Sciences Forschungsverbund, University of Vienna, Vienna, Austria

**Keywords:** Element cycles, Stable isotope analysis

## Abstract

We identify a major sulfur isotope excursion in Eurasian faunal bone collagen from the last 50,000 years, here termed the Late Pleniglacial Sulfur Excursion. Our analysis suggests this is linked to changing permafrost conditions, presenting the utility of faunal collagen δ^34^S as a proxy for permafrost dynamics, a critical component of the global carbon cycle. Our findings complicate the use of archaeological faunal sulfur isotopes for mobility and palaeodietary studies.

## Abstract (web summary)

Sulfur isotope excursions in skeletal collagen samples from Eurasia suggest rapidly changing biogeochemical processes across the region from about 30,000 to 15,000 years ago, potentially related to changing permafrost conditions.

## Main

Over the past two decades, sulfur isotope ratios (δ^34^S) in plant, animal and human tissues have been increasingly used to explore food provenance, present and past diets and human and animal mobility. Most studies use sulfur isotopes as a geolocator, leveraging the spatial variability observed in plant sulfur isotope values, reflecting those of bioavailable sulfur. This variability arises because soil sulfur is primarily derived from mineral weathering of parent bedrock, the δ^34^S of which varies by rock type^1^. Additionally, the atmosphere contributes sulfur to soils (via dry deposition, SO_4_^2−^ aerosols or wet deposition of SO_4_^2−^), although pre-industrial atmospheric inputs contributed <10% of total soil S, excepting narrow zones of strong coastal seawater sulfate spray influence^1,2^. Recent studies suggest that waterlogged soil conditions may result in distinct bioavailable δ^34^S values^3,4^. Minimal fractionation is seen in organic-bound sulfur as it is passed along the food chain (Δ^34^S tissue-diet ≈ 0 ‰ (refs. ^5,6^)), so animal δ^34^S values closely reflect those of the bioavailable δ^34^S at the base of their food chain^7,8^. Thus, animal δ^34^S values have been used to determine origin or mobility/migratory behaviours. Others use sulfur isotopes as a (palaeo)dietary indicator, as marine resources have high and relatively homogeneous δ^34^S values (about 20‰), whereas terrestrial δ^34^S tends to be lower and more variable^8^. Overall, animal and human δ^34^S values are commonly interpreted as reflecting one or more stable sources, uninfluenced by environmental change, whereas a few studies argue that archaeological δ^34^S values reflect locally variable hydrological dynamics^9–11^.

**Fig. 1:**
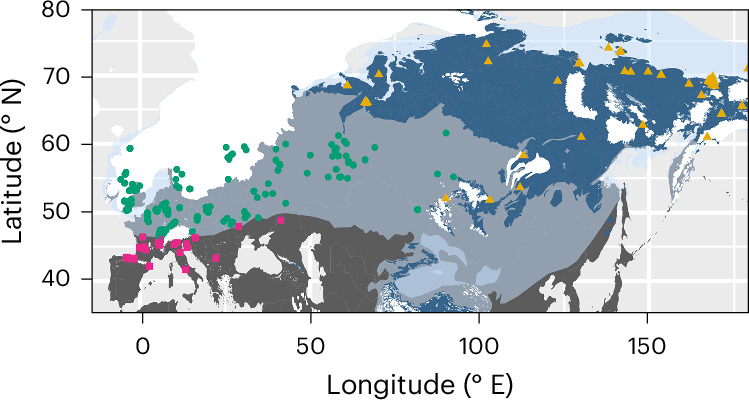
Geographical distribution of the faunal samples. Pale blue area indicates approximate zone of continuous permafrost at the LGM^20^. White area indicates LGM extent of ice sheets and glaciers^40^. Dark blue area indicates zone of present-day continuous and discontinuous permafrost distribution^41^. Pink squares: samples collected from regions where no permafrost was present during the last 50,000 years. Green circles: samples collected from areas that either had permafrost present or were under ice sheets/alpine glaciers at the LGM but where permafrost/ice sheets/alpine glaciers are absent today. Yellow triangles: samples collected from regions in which permafrost has been present throughout the past 50,000 years.

**Fig. 2:**
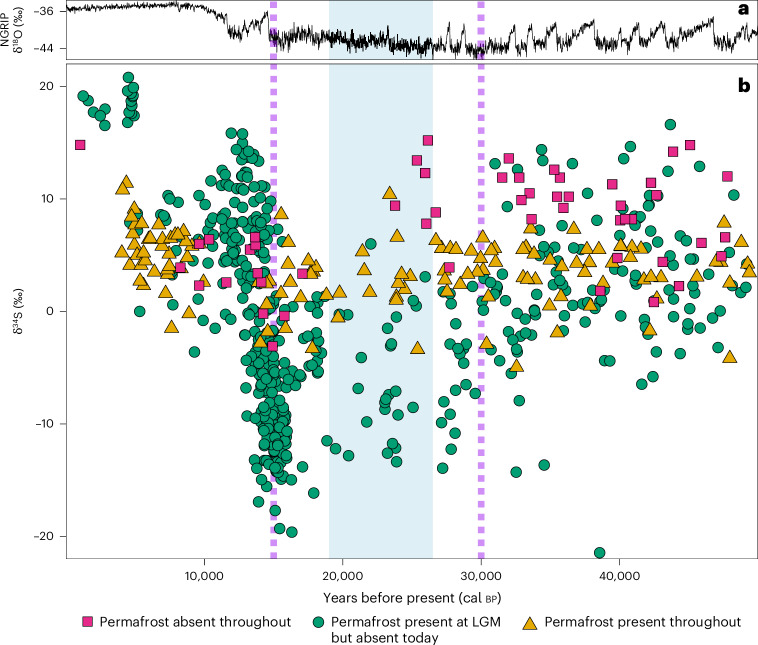
Faunal collagen δ^34^S values from Eurasia through the Late Pleistocene and Holocene. Each data point represents a single animal specimen, which has been directly radiocarbon dated. **a**, The North Greenland Ice Core Project (NGRIP) oxygen isotope record^42^, a proxy for global temperature. **b**, Faunal collagen δ^34^S values. Shaded blue area indicates approximate duration of the LGM. The dashed purple lines indicate approximate timing of continuous permafrost development (about 30 kyr bp) and thaw (about 15 kyr bp) in western Eurasia. Pink squares: samples collected from regions where no permafrost was present during the last 50,000 years. Green circles: samples collected from areas that either had permafrost present at the LGM or were under ice sheets/glaciers at the LGM but where permafrost/ice is absent today. Yellow triangles: samples collected from regions in which permafrost has been present throughout the past 50,000 years.

**Fig. 3:**
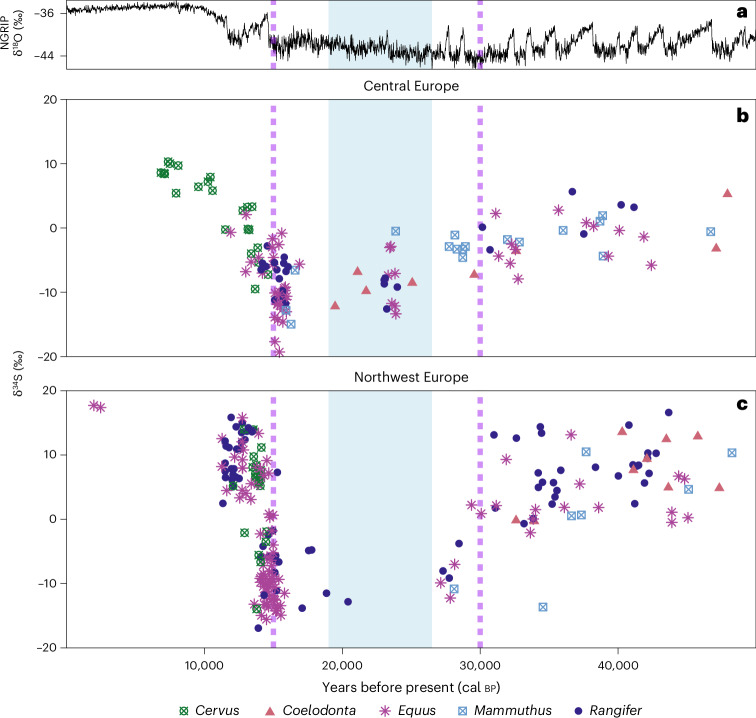
δ^34^S values of radiocarbon-dated herbivores from central and northwest Europe for the five most abundant taxa in both regions, *Cervus elaphus, Coelodonta antiquitatis, Equus sp., Mammuthus primigenius* and *Rangifer tarandus*. **a**, The Greenland ice-core oxygen isotope record, a proxy for global temperature^42^. **b**,**c**, δ^34^S values of radiocarbon-dated herbivores from central and northwest Europe (46.5° N to 54° N, 6° E to 21° E) (**b**) and northwest Europe (50° N to 60° N, 10° E to 6° W) (**c**). Shaded blue area indicates approximate duration of the LGM. The dashed purple lines indicate approximate timing of continuous permafrost development (about 30 kyr bp) and widespread thaw (about 15 kyr bp) in western Eurasia.

Here we report results of 796 δ^34^S isotope and 691 accelerator mass spectrometry (AMS) radiocarbon analyses from Late Pleistocene and Holocene fauna from Eurasia (Figs. 1 and 2, Supplementary Discussion 1.1 and Supplementary Data 1). One hundred and five samples come from contexts previously AMS dated. Our results show a high-magnitude excursion in faunal δ^34^S isotope values between approximately 30 and 15 thousand years (kyr) before present (bp) in some regions of Eurasia (Fig. 2 and Supplementary Fig. 1). This period corresponds to the latter part of the last ice age across much of Marine Isotope Stage 2 (about 29–11.7 kyr bp), including the Last Glacial Maximum (LGM, about 26.5–19 kyr bp). This excursion, which we name the Late Pleniglacial Sulfur Excursion (LPSE), is particularly pronounced in regions where we have good temporal coverage within a discrete geographic area, such as in Britain and Belgium, and is also evident in other regions, such as central Europe north of the Alps (Fig. 3 and Supplementary Fig. 2). However, the temporal and spatial coverage prevent us from determining whether the LPSE is time transgressive across this region. The LPSE occurs across multiple species with differing dietary niches and mobility behaviours (Fig. 2). The LPSE magnitude is substantial (up to 35‰), more than double that typically considered to indicate location-based differences^12^. This suggests that underlying continental-scale processes substantially impacted the terrestrial sulfur cycle during the Late Pleistocene.

Our Eurasian samples span the last 50 kyr (Fig. 1). The climate between about 50 and 28 kyr bp (Middle Pleniglacial) featured millennial-scale oscillations between cold stadial and mild interstadial states and fluctuating sea levels, superimposed on a long-term trend towards colder conditions and lower sea levels^13,14^. The Middle Pleniglacial environment across northwest and central Europe was wet and densely vegetated, with long periods of seasonal frost, and some discontinuous permafrost^15,16^. The onset of the Late Pleniglacial (about 28–14.7 kyr bp) saw the major expansion of European ice sheets^17,18^. Maximum ice-sheet extent occurred during the LGM, when sea levels were about 130 m lower than today^19^. Continuous permafrost (ground that remains ≤0 °C for at least two consecutive years) was widespread across northern Eurasia at this time^20^. Between about 25 and 22 kyr bp, increasing aridity in northern Europe induced widespread fluvio–aeolian deposition in river valleys^21^, with maximum aridity between about 17 and 15 kyr bp (ref. ^21^). During continental deglaciation, sea levels rose slowly from about 19 kyr bp, then more rapidly from about 16 to 12.5 kyr bp (ref. ^14^). Widespread thaw of permafrost in NW Europe was instigated by slight warming at about 17–15 kyr bp then rapid warming at the start of the Late Glacial Interstadial about 14.7–12.9 kyr bp (ref. ^21^). There was a brief return to colder conditions (Younger Dryas/Greenland Stadial 1, about 12.9–11.7 kyr bp) before the present interglacial, the Holocene. After the Younger Dryas, sea-level rise was rapid, approaching present-day levels around 7 kyr bp (ref. ^14^).

Variations in herbivore collagen δ^34^S are typically interpreted as reflecting differences in animal spatial ecology, driven by differences in the δ^34^S of consumed plants with varying underlying geology and/or distance from coast. However, our data analysis shows that animal spatial ecology is not the primary driver of the LPSE. There are many potential influences such as changes in atmospheric sulfur source (particularly loess and sea-spray) linked to sea-level variations, sulfur emissions from volcanic activity, changes in bedrock weathering rates linked to glaciation, temperature and precipitation changes (Supplementary Discussion 1.2 provides detailed discussion). However, our multivariate analysis concludes that changing near-surface permafrost conditions are the most plausible driver of the observed excursion (Supplementary Discussion 1.4).

Permafrost currently underlies substantial areas of Alaska, Canada, Siberia and Greenland and was present throughout the Late Pleistocene. During the LGM, permafrost expanded across northern Eurasia^20^. Its maximum extent has been established via mapping of permafrost-related geomorphological features^22^. Notably, our faunal δ^34^S values differ substantially between regions with different permafrost histories (Supplementary Discussion 1.4, Supplementary Tables 6 and 7 and Supplementary Fig. 11). The LPSE is observed exclusively in regions where permafrost (or ice sheets/alpine glaciers) was present during the LGM but are now permafrost free (Fig. 2) and coincides with the timing of regional permafrost development and thaw inferred from local geomorphological evidence^22,23^ and Greenland ice cores^24^ (Supplementary Fig. 1). The LPSE is not observed in regions where permafrost is present today and persisted throughout the Late Pleistocene (Figs. 1 and 2), nor in regions where permafrost was not present over the last 50 kyr (Supplementary Fig. 1).

We highlight two processes associated with permafrost thaw that may have driven the shift to lighter δ^34^S during the LPSE, both of which would have impacted plant (and therefore animal) δ^34^S: inputs from newly thawed substrate generated by active-layer deepening and the development of anoxic wetlands due to impeded drainage.

Permafrost growth impedes weathering of underlying bedrock and sediments. Active-layer deepening and thawing therefore increase the input of sedimentary sulfides and enhance sulfide oxidation^25^, transferring the negative sulfide δ^34^S signature to soils. Permafrost development also substantially influences hydrology, impeding drainage and confining soil water to the active layer, which can induce periodic waterlogged, anoxic soil conditions. Such conditions alter soil redox status, enhancing sulfide production via dissimilatory sulfate reduction (DSR) in bacteria and archaea^26^, a process that can produce large (–46 to –40‰) isotopic fractionation^27^. These low-sulfide δ^34^S values are inherited by plants after re-oxidation to sulfate^28,29^. Microbial DSR readily occurs in cold regions^30–33^, including areas of modern permafrost thaw^34,35^. Sufficiently cold temperatures, however, suppress the extent of DSR^36^ and inhibit weathering, potentially explaining the lack of LPSE in regions with stable permafrost across the last 50,000 years (Supplementary Fig. 1 and Supplementary Data 1). It follows, therefore, that the LPSE is only observed in those lower-latitude regions subject to extensive climatic, environmental and permafrost perturbations. Intermediate temperatures (Supplementary Fig. 1 and Supplementary Data 1) and young, epigenetic permafrost^37^ would have enhanced weathering rates, sulfur availability and DSR throughout the Late Pleistocene, followed by extensive thaw after the LGM, producing the lowest δ^34^S values.

Further investigation into the spatio-temporal extent and continuity of these processes, including whether the LPSE is observed in other regions such as North America, is needed to assess the degree to which permafrost change drives the LPSE.

Modern permafrost is a major reservoir of organic carbon, which is being released to the atmosphere as CO_2_ and methane as the Arctic warms at twice the global average rate^38^, accelerating warming^39^. We can investigate permafrost sensitivity to climate shifts by studying the relationship between past permafrost conditions and palaeoclimate change. However, this is hindered by limited data on the timing of permafrost growth and thaw. The geomorphological features used to assess past permafrost extent are often difficult to date^18^. Our results show that sulfur isotope analysis of faunal samples could provide high-resolution records of past permafrost change because the preserved isotopic signature of bone collagen represents only a few years to decades of the animal’s life, restricted to its home range, and bone collagen from the last about 50,000 years is directly dateable through radiocarbon methods with good preservation potential. The spatio-temporal patterns of these data allow insights into permafrost development and degradation at local to continental scales. More broadly, our findings indicate animal δ^34^S values can reflect changes in local hydrology and loess deposition, complicating the use of δ^34^S as a provenancing tool for food origin, animal migration and archaeological research. This issue will not arise in faunal datasets of similar age, with stable permafrost, hydrology and loess deposition. Furthermore, these findings support the utility of sulfur isotopes to examine wetland habitat use by people and animals in modern and archaeological contexts.

## Methods

Our dataset comprises 796 δ^34^S isotope analyses (510 new and 286 previously published δ^34^S values (Supplementary Data 1). New δ^34^S analyses were undertaken on collagen extracted from 395 bones and 115 teeth sourced from 68 archaeological/palaeontological sites (*n* = 1 to 56 samples per site). Of our 510 new δ^34^S analyses, 221 were carried out on collagen previously extracted at Oxford Radiocarbon Accelerator Unit (ORAU) for radiocarbon dating; 221 were carried out on collagen previously extracted for our previous studies^43–50^ and 68 on collagen extracted for this study. The method of collagen extraction for each sample is given in Supplementary Data 1 and fully described in our recent study^44^. Only data with C/S and N/S ratios indicative of well-preserved collagen were compiled from the literature^51,52^. Genus/species represented in our dataset include *Bos/Bison* sp., *Capreolus capreolus*, *Cervus elaphus*, *Alces alces*, *Coelodonta antiquitatis*, *Equus* sp., *Mammuthus primigenius*, *Megaloceros giganteus*, *Ovibos moschatus*, *Ovis aries*, *Rangifer tarandus*, *Rupicapra*, *Saiga tatarica* and *Sus* (Supplementary Data 1). New AMS determinations were undertaken at ORAU for 25 samples, and 666 of the samples had previously been radiocarbon dated by AMS. Sample details and preparation methods are given in Supplementary Data 1 and Supplementary Discussion 1. Sulfur isotope ratios were determined on the extracted collagen using a Delta V Advantage continuous-flow isotope ratio mass spectrometer coupled via a ConfloIV to an IsoLink elemental analyser (Thermo Scientific) at the Scottish Universities Environmental Research Centre. Samples were weighed into tin capsules (~1.2–1.5 mg) and combusted in the presence of oxygen in a single reactor containing tungstic oxide and copper wires at 1,020 °C to produce SO_2_. A magnesium perchlorate trap was used to eliminate water produced during the combustion process, and the gas was separated in a gas chromatography column heated between 70 °C and 240 °C. Helium was used as a carrier gas throughout the procedure. SO_2_ entered the mass spectrometer via an open split arrangement within the ConfloIV and was analysed against a reference gas. Samples were analysed in duplicate, with the exception of 16 samples for which there was only sufficient collagen for a single analysis. For every ten unknown archaeological samples, either three gelatine-based in-house standards (SAG: δ^34^S_VCTD_ = −10.1 ± 0.1‰, MAG: δ^34^S_VCTD_ = 1.4 ± 0.1‰ and MSAG: δ^34^S_VCTD_ = 11.1 ± 0.1‰) or two gelatine-based in-house standards (SAG2B: δ^34^S_VCTD_ = −9.5 ± 0.1‰ and MSAG2: δ^34^S_VCTD_ = 11.5 ± 0.1‰), which were calibrated to the International Atomic Energy Agency (IAEA) reference materials IAEA-S-1 (silver sulfide, δ^34^S_VCTD_ = −0.3 ± 0.2‰), IAEA-S-2 (silver sulfide, δ^34^S_VCTD_ = 22.7 ± 0.2‰), IAEA-SO-5 (barium sulfate, δ^34^S_VCTD_ = 0.5 ± 0.2‰) and IAEA-SO-6 (barium sulfate, δ^34^S_VCTD_ = −34.1 ± 0.2‰) were used to normalize the δ^34^S_VCTD_ values. Results are reported as per mille (‰) relative to the internationally accepted standard Vienna Canyon Diablo Troilite (VCDT). Normalization was checked using United States Geological Survey (USGS) reference material USGS43 (Indian human hair: δ^34^S_VCTD_ = 10.5 ± 0.2‰) or the well-characterized Elemental Microanalysis Isotope Ratio Mass Spectrometry fish gelatin standard B2215 (δ^34^S_VCTD_ = 1.2 ± 0.2‰), and long-term precision was determined to ±0.4‰ for δ^34^S based on repeated measurements of an Iron Age in-house horse bone collagen standard (DHB2019: δ^34^S_VCTD_ = 9.5 ± 0.2‰, *n* = 1,246). All our samples had C/S and N/S ratios (306–898 and 100–284) within the quality range indicative of well-preserved collagen for sulfur isotope analysis^51^. Statistical analyses were undertaken in R version 4.3.0 to explore potential drivers of the LPSE. Parameters considered include underlying geology, proximity to loessic deposits, proximity to palaeocoastlines, proximity to palaeo-ice sheets, modelled mean annual temperature and precipitation, proximity to present-day and LGM permafrost. Data were investigated using Kruskal–Wallis tests and factor analysis of mixed data, which considers continuous and categorical variables within the same model (full details of statistical analyses are given in Supplementary Data 1). A hierarchical cluster analysis of the three most important components from the factor analysis of mixed data analysis was conducted (Supplementary Fig. 13), identifying subgroups in the data that share similar characteristics, exploring variables contributing to the δ^34^S trends. The identified clusters were then considered in relation to the temporal trend observed in the δ^34^S data.

## Online content

Any methods, additional references, Nature Portfolio reporting summaries, source data, extended data, supplementary information, acknowledgements, peer review information; details of author contributions and competing interests; and statements of data and code availability are available at 10.1038/s41561-025-01760-x.

## Supplementary information

Supplementary Information Supplementary Discussion 1.1–1.4 and Figs. 1–17.

Supplementary Data 1 Primary dataset.

Supplementary Tables 1–9 Source data for Supplementary Discussion 1.4.

## Data Availability

All data are available at 10.5522/04/28677713 (ref. ^52^) and in the Supplementary Information accompanying this article.
