# Supplementary material for: Major excursions in sulfur isotopes linked to permafrost change in Eurasia during the last 50,000 years
Source: Nat Geosci. 2025 Aug 1;18(10):961–5. doi: 10.1038/s41561-025-01760-x (PMC12510881; doi:10.1038/s41561-025-01760-x)
Supplement: Supplementary file 1 — Supplementary Discussion 1.1–1.4 and Figs. 1–17. [file 41561_2025_1760_MOESM1_ESM.pdf]

nature geoscience

Supplementary information

https://doi.org/10.1038/s41561-025-01760-x

Major excursions in sulfur isotopes linked to permafrost change in Eurasia during the last 50,000 years

In the format provided by the authors and unedited

S1: Supplementary information 1:

S1.1: Alternate Figures 2
S1.2: Alternative hypotheses for drivers of the LPSE 5
S1.2.1: Animal origin / mobility 5
S1.2.2: Change in sulfate source linked to sea levels 5
S1.2.3: Sulfur emissions from volcanic activity 6
S1.2.4: Change in bedrock weathering rates linked to glaciation 7
S1.2.5: Climate change 8
S1.3: Additional discussion of sulfur biogeochemical cycling and permafrost 8
S1.4: Statistical investigation of potential drivers of the LPSE 10
S1.4.1: Bedrock geology 10
S1.4.2: Unconsolidated sediment 13
S1.4.3: Palaeoclimate, hydrosphere and cryosphere 17
S1.4.4: Permafrost 20
S1.4.5: Multivariate Analysis 22
References 28

Literature referenced herein is numbered independent of the main article and references are listed at the end of this document.

S1.1: Alternate Figures

Figure S1 (next page): Faunal $\delta^{34}S$ values from Eurasia through the Late Pleistocene and Holocene. Each data point represents a single animal specimen which has been directly radiocarbon dated. Panel A shows the Greenland ice-core oxygen isotope record, a proxy for global temperature[1]. Panel B shows samples collected from regions where no permafrost was present during the last 50,000 years. Panel C shows samples collected from areas that either had permafrost present at the LGM or were under ice sheets /alpine glaciers at the LGM, but where permafrost/ice sheets/alpine glaciers are absent today. Panel D shows samples collected from regions in which permafrost has been present throughout the past 50,000 years. Shaded blue area indicates approximate duration of the Last Glacial Maximum. The dashed purple lines indicate the approximate timing of continuous permafrost development (c. 30 kyr BP) and thaw (c. 15 kyr BP) in western Eurasia. The development of continuous permafrost there at c. 30 kyr BP is inferred from a synthesis study that found the majority of reported Pleistocene ice-wedge pseudomorphs developed in Europe between 31 and 24 kyr BP[2] and the time of transition from milder and more variable environments of MIS 3 to more uniformly cold environments of MIS 2, as shown by the NGRIP oxygen-isotope record from Greenland, upwind of Europe[3]. The thaw of near-surface continuous permafrost at c. 15 kyr BP is based partly on geological evidence that large ice- or sand wedges actively grew in Europe until 18–17 kyr BP[2] and the abrupt, major phase of climate warming at the onset of the Bolling–Allerod period 14.7 kyr BP[3]. Gradual climate warming post-LGM (i.e., post 19 kyr BP) would likely have taken some millennia to thaw permafrost completely (due to thermal inertia from latent heat effects of ground ice), and so we consider 15 kyr BP a reasonable approximation of the time when near-surface continuous permafrost had degraded. N.B. the slightly lower $\delta^{34}S$ (0‰ to -4‰) values around 15 kyr BP in panel B, notably, are all located on the permafrost margin (Fig 1.) and given there is uncertainty associated past permafrost extent, are not guaranteed to be unaffected by changing permafrost conditions.

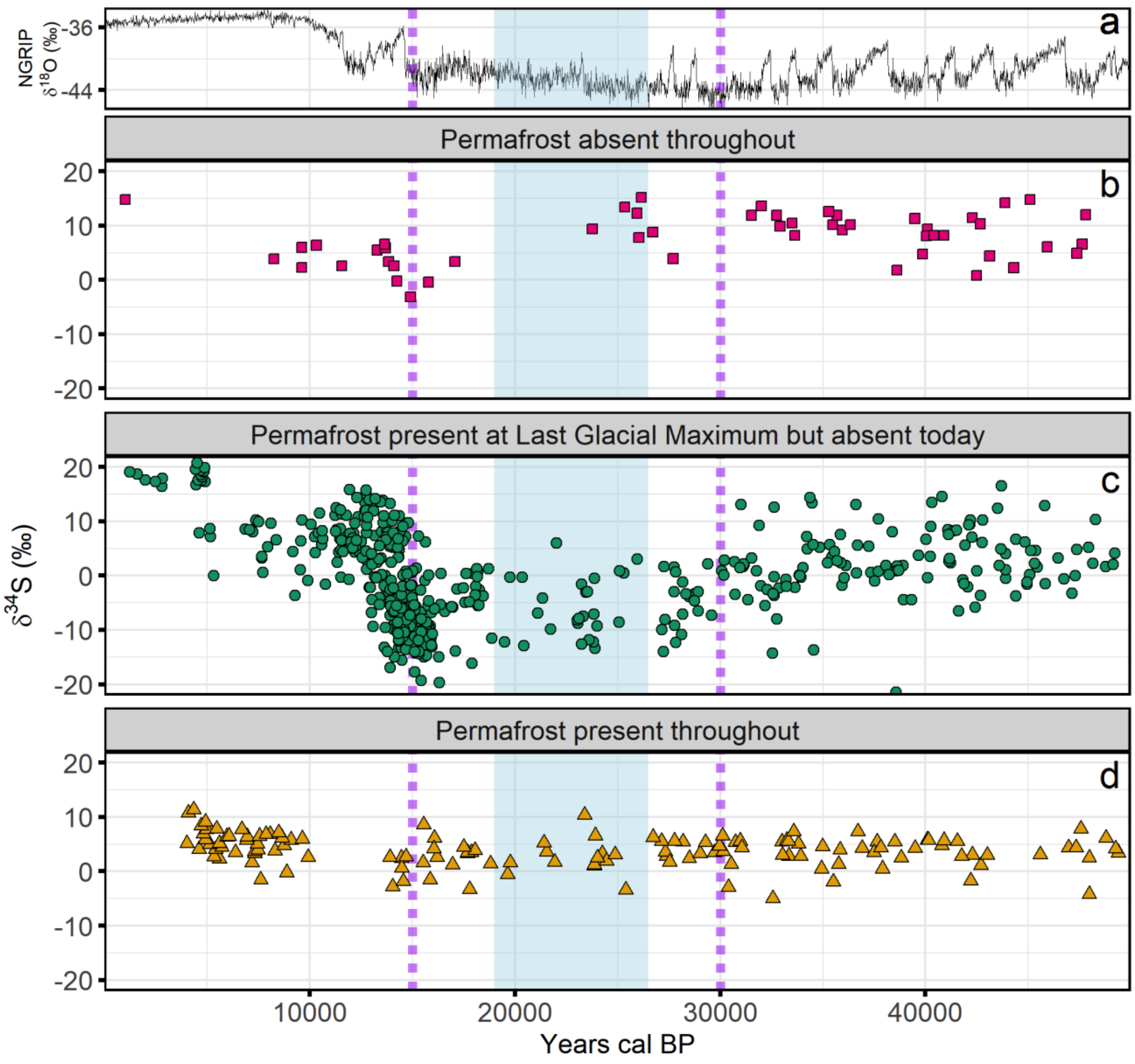

NGRIP δ18O (‰)
-36
-44
a
Permafrost absent throughout
b
Permafrost present at Last Glacial Maximum but absent today
c
δ34S (‰)
Permafrost present throughout
d
20
10
0
-10
-20
10000
20000
30000
40000
Years cal BP

Figure S2: Box plots of faunal δ^{34}S values of context dated fauna (*Alces alces, Bos/bison sp., Equus sp., Mammuthus primigenius, Ovis aries, Rangifer tarandus, Sus scrofa*) from Czechia (central Europe). This shows the LPSE is clearly observed in central Europe. Box indicates inter-quartile range (IQR), line in box indicates median, whiskers indicate 1.5 times the IQR, and points show individual data.

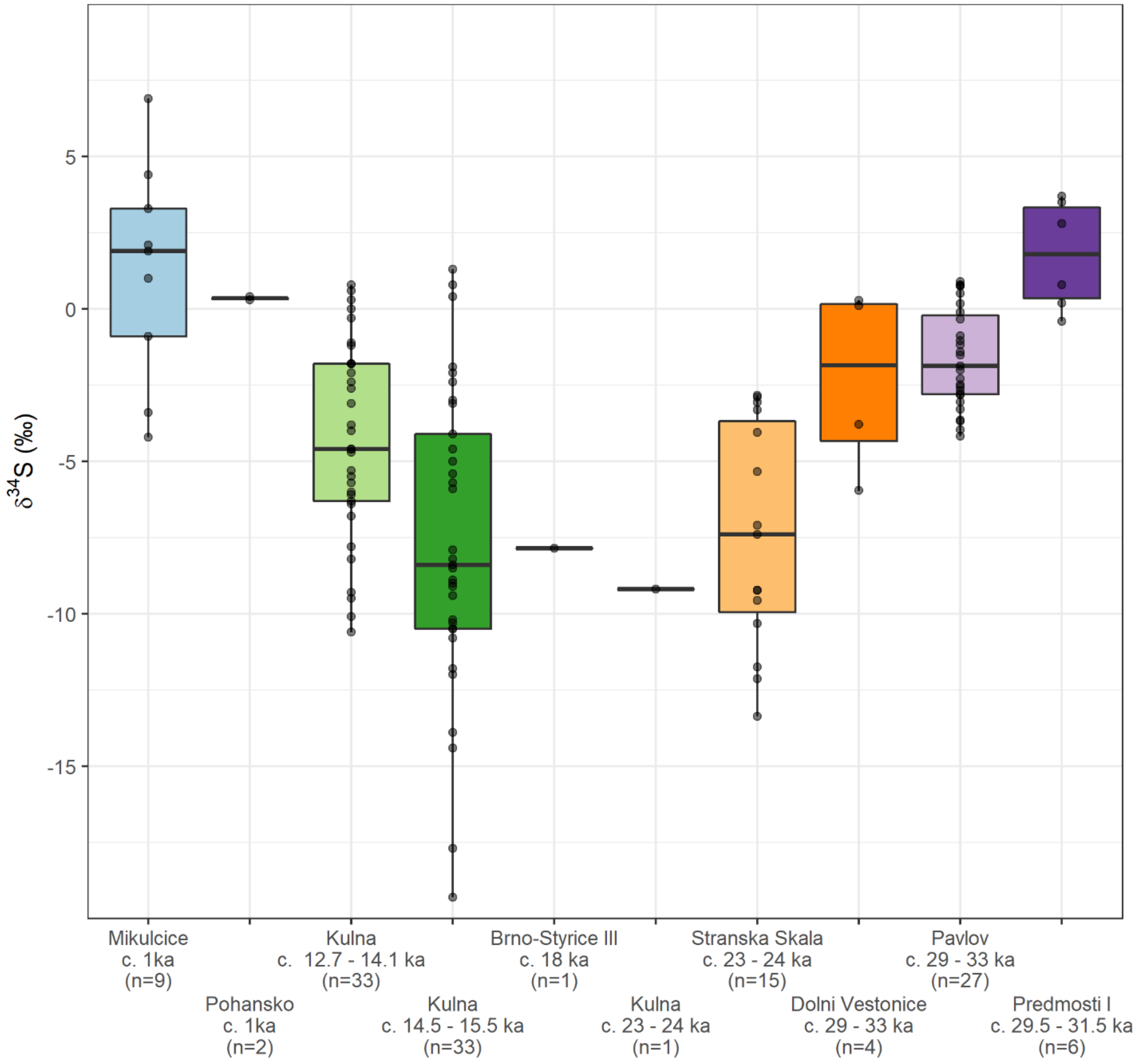

S1.2: Alternative hypotheses for drivers of the LPSE

S1.2.1: Animal origin / mobility

Variations in animal bone collagen $\delta^{34}S$ values are typically interpreted as animals feeding in different locations with different bioavailable ^{34}S due to underlying geology and/or distance from coast. However, the LPSE cannot be explained by changes in animal feeding locations. First, our samples were recovered from a variety of geological contexts, yet we see no spatial correlation between faunal $\delta^{34}S$ values and geology (Fig S3, Table S1, Table S2). The low $\delta^{34}S$ values are present across a range of bedrock and sediment types (Figs S3, S6, S8) and the excursion to lower values is seen on all rock ages and sediment types (Figs S4, S5, S7, S9), suggesting that proximity to specific rocks and sediments is not the primary driver of the temporal variation in ecosystem $\delta^{34}S$. Second, the same temporal trend is observed within multiple discrete geographic locations in several species with very different feeding and mobility behaviours, demonstrating the temporal $\delta^{34}S$ trend is not driven by species-specific behaviours (Fig. 3).

S1.2.2: Change in sulfate source linked to sea levels

Changing sea levels during the last 50 kyr potentially impacted the terrestrial sulfur cycle in two ways. First, atmospheric transport and deposition of ^{34}S-enriched sulfur from the marine to terrestrial environments via sea spray impacts terrestrial soil, plant, and animal $\delta^{34}S$ values. This sea-spray effect most greatly influences terrestrial $\delta^{34}S$ values within c. 30 km of the coast[4], although smaller amounts of marine sulfates can be deposited farther inland[5,6]. As sea level fell some regions became more distant from the coast, and vice versa as sea level rose[7,8], thus potentially impacting ecosystem $\delta^{34}S$ values. However, we find no clear correlation between proximity to time-matched palaeoshorelines and faunal $\delta^{34}S$ values (Fig. S10 (d)). While the highest faunal $\delta^{34}S$ values (>15‰) appear to only occur within 250 km of the coast, values in this region and farther inland range from –15 to +20‰. Furthermore, the LPSE is present in many locations that remained many hundreds of km from the coast throughout the Late Pleistocene (e.g. central Europe) (Fig. 3; Fig S2). Thus, variation in the quantity of marine-derived sulfur due to sea-level change is unlikely to be the primary driver of the LPSE.

Second, as Late Pleistocene sea level fell, newly exposed land became a potential new source of sulfate through aeolian sediment transportation and deposition of loess, sandy loess, and aeolian sand. If these sediments were ^{34}S-depleted, this could result in a trend to lower soil, plant and faunal δ^{34}S values in the Late Pleniglacial. Continental deglaciation after the LGM resulted in flooding of the extensive areas of the North Sea Basin[9], cutting off a potential aeolian sediment source. An increase in terrestrial aeolian sediments has been postulated to explain the depleted sulfur isotope ratios observed in the Antarctic Dome Fuji ice core during the LGM compared to those seen during the Holocene[10]. However, the values observed are all above 10‰ and the shift in values between the LGM and Holocene is only ~6 ‰, thus not of the magnitude seen in the faunal isotope values. We do see a significant difference in faunal δ^{34}S between samples recovered from within 10 km of loess sediments (i.e. animals most likely to have eaten plants grown on loessic sediments) and those farther away from loessic deposits (Fig. S8, Table S4, Table S5). Yet temporal trends in δ^{34}S show that the excursion to lower δ^{34}S during the LGM and Late Glacial occurs in samples within 10 km of loess as well as those more than 50 km away from loess (Fig. S9). This suggests that proximity to loess sediments is not the primary driver of the LPSE, but a confounding factor. It is important to highlight, however, that Pleniglacial aeolian deposits have been eroded away and so the present-day distribution is more limited compared to during the Late Pleniglacial. In addition, aeolian deposition occurred in northwest Europe prior to the onset of the LPSE, although a substantial increase in sedimentation did occur between c. 32 and 15 kyr BP[11]. Furthermore, the inundation of the North Sea Basin was a prolonged event, mainly occurring between 12 to 6 kyr BP[12], with different areas inundated at different stages[13]. This gradual cutting off of potential sulfate sources from North Sea sediments is inconsistent with the rapid rise in faunal δ^{34}S values seen at the end of the LPSE.

S1.2.3: Sulfur emissions from volcanic activity

Volcanoes release sulfur into the atmosphere through passive degassing or eruptive activity. Wind and rain disperse volcanic aerosols and dust, which eventually settle on land surfaces and potentially affect ecosystem δ^{34}S values. Volcanic sulfate aerosols have a short residence time, lasting from weeks in the troposphere to a

couple of years in the stratosphere[14,15]. As a result, they quickly deposit on land. To be the main driver of the LPSE, volcanic activity would need to be substantial and sustained during this period. However, there was no significant difference in volcanic sulfate deposits before and after approximately 30 ka BP[16], when the substantial shift in faunal $\delta^{34}S$ occurred. Additionally, for volcanic activity to be the primary driver of the LPSE, the volcanic sulfur emissions would have had to be ^{34}S-depleted. However, $\delta^{34}S$ values of volcanic gas and dust are wide ranging, centring on 0‰[17]. The melting of glacial ice during the last deglaciation (c. 16–9 ka BP) triggered increased volcanic activity due to pressure unloading, as evidenced by higher levels of volcanic sulfates in the Greenland ice cores[18,19]. It is possible that the post 15 ka BP rebound in faunal $\delta^{34}S$ could be partly driven by volcanic activity in some areas, but not all. For example, the most significant Late Glacial volcanic eruption in the region where the LPSE is detected came from the Laacher See volcano (western Germany). The sulfur-rich eruption occurred c. 13,000 years ago and tephra / cryotephra evidence shows ash from the eruption was carried up to 1800 km (with the amount of ash deposited decreasing with distance)[20,21]. However, this ash was only carried in three directions, to the northeast, south and west, but not to Britain where the rebound in faunal $\delta^{34}S$ is particularly visible[21].

S1.2.4: Change in bedrock weathering rates linked to glaciation

The terrestrial sulfur cycle is influenced by bedrock weathering. Changes in the rate of weathering, linked to the melting of the British–Irish and Fennoscandian ice sheets and alpine glaciers, likely impacted bioavailable sulfur isotope values. The exposure of glacial sediments during glacial melt may have caused the oxidation of primary sulfide minerals, resulting in a temporary increase in availability of ^{34}S-depleted sulfate. We observe no relationship between proximity to palaeo-icesheet margins and faunal $\delta^{34}S$ values, although low $\delta^{34}S$ values appear to only occur within c. 1000 km of ice sheets (Figure S10 (c)). Proximity to palaeo-icesheets is unlikely to be the primary driver of the LPSE as we observe the temporal changes in faunal $\delta^{34}S$ values in regions several hundred kilometres beyond the maximum extent of the Late Pleistocene glacial limits[22] (Fig. 3 and Fig. S2).

S1.2.5: Climate change

Temperature and precipitation amounts have substantially varied over the period covered in this study[23]. Both have the potential to impact the biogeochemistry of sulfur, through influencing weathering rates and soil hydrological conditions. Precipitation does not appear to be the primary driver of the LPSE as we see no clear correlation between modelled mean annual palaeoprecipitation[24] and faunal $\delta^{34}S$ values (Fig S10b). Likewise, there is no relationship between modelled mean annual air temperature (MAAT)[24] and faunal $\delta^{34}S$ values, though low $\delta^{34}S$ values (<–8‰) occur almost exclusively in environments where MAAT was between c. 0 and 7ºC (Fig S10a). Faunal $\delta^{34}S$ values above –8‰ occur across the full MAAT range from c. –30°C to c. 10°C. It is notable that for MAATs between 0°C and 7°C permafrost would generally thaw.

S1.3: Additional discussion of sulfur biogeochemical cycling and permafrost

Few studies, to date, have examined the impact of permafrost growth and thaw on sulfur cycling. Those available show several processes during permafrost development and thaw which can influence sulfur cycling. Establishment of permafrost limits the weathering of bedrock, suppressing the input of sulfide minerals to the bioavailable reservoir. Conversely, thawing of permafrost enables weathering of sulfides and oxidation to bioavailable sulfate[25]. Depleted bedrock sulfide $\delta^{34}S$ (–32.2‰) and stream water sulfate $\delta^{34}S$ values (–13.1‰ to –26.2‰) have been reported from retrogressive permafrost thaw slumps in Canada[25]. Permafrost growth also has a significant impact on hydrological processes. Liquid soil water is only present in the active layer, so drainage is significantly limited, often leading to waterlogging and establishment of zones of anoxia in the active layer. Similarly, thaw of ice-rich permafrost and the development of thermokarst may also initially result in waterlogging. These changes in the local redox state lead to the production and accumulation of sulfides as the end product of dissimilatory sulfate reduction (DSR) by bacteria and archaea which use sulfate as an electron recipient for redox reactions[26]. This process discriminates strongly against ^{34}S [27] and can therefore produce significant (–46 to –40‰) isotopic fractionation in the resultant sulfide. Some of these ^{34}S-depleted sulfides are re-oxidised to sulfate, which plants can directly take up. As minimal isotopic fractionation occurs during re-oxidisation, the sulfate produced is isotopically light, resulting in very low plant $\delta^{34}S$ values[28,29]. In addition, the ^{34}S-depleted sulfides can be taken up directly by certain plants which can tolerate sulfide toxicity

or which can re-oxidise sulfide to SO_4^{2-} through transporting oxygen to their roots[30,31]. Many studies record DSR in modern subarctic environments[32-35]. A study on pore water sulfate $\delta^{34}S$ in saturated permafrost ice-wedge polygons in Svalbard reported that DSR preferentially reduced ^{34}S, with product H_2S subsequently incorporated into organic matter[36]. DSR and sulfide oxidation have been shown to impact sulfate $\delta^{34}S$ values (–19‰ to +10‰) in streams and groundwater in Yukon Territory, Canada, where permafrost is thawing[33]. Furthermore, analyses of sulfur isotopes of water and sediments from a major tributary of the Yukon River draining discontinuous permafrost regions showed evidence of DSR within the floodplain, and extensive sulfide oxidation[37].

We do not see the LPSE where permafrost existed from the Late Pleistocene to the present. Permafrost in these regions has commonly been present for hundreds of thousands of years and widely formed syngenetically in freshly deposited sediment, with the base of the active layer aggrading upwards alongside continued cold-climate sedimentation[38]. Climate and environmental conditions in these regions over the timescale of this study have been relatively stable. furthermore, the modelled mean annual air temperatures (MAATs) for the sample locations in this region are between –30°C and –12°C (S1.4, SD1). Such low temperatures severely limit bedrock weathering rates, sulfide inputs, sulfur availability and active-layer depth. Some sulfate-reducing bacteria and archaea species that undertake DSR may be inactive at such low temperatures, and, where active, result in more limited fractionation at the lower extreme of their temperature range[39].

So far, the LPSE is only observed in regions where permafrost developed then thawed during the last 50,000 years. These regions were subject to extensive climatic and environmental change during this period. This permafrost primarily formed epigenetically, from the ground surface downwards in soils or sediments that had existed for hundreds or thousands of years before perennial freezing started[38]. The trend to lower $\delta^{34}S$ values through the LGM occurs in regions where the modelled MAATs for the sample locations are between –13°C and 5°C (S1.4, SD1). Under such conditions the weathering rates, sulfur availability and DSR are likely to have exceeded those in regions where permafrost was present throughout the Late Pleistocene to the present day. The lowest $\delta^{34}S$ values appear after the LGM as permafrost degraded. Such low values may be explained by the input of bedrock sulfide due to enhanced weathering, and enhanced DSR due to increasing temperatures and soil saturation amidst thawing permafrost and icesheets and wetter climatic conditions. The return to higher $\delta^{34}S$ values towards the end of the Pleistocene is consistent with the return of well-drained soils

(aerobic conditions) and therefore less DSR, along with a decline in input of sulfur from bedrock weathering (as the deeper substrate reservoir becomes exhausted).

Whilst many processes during permafrost growth and thaw likely impact sulfur cycling, limited investigations make it difficult to determine the balance between these different processes, whether one is dominant, or if they synergistically impact the sulfur cycle, resulting in the observed trends in faunal $\delta^{34}S$ values. Ongoing research into sulfur isotopes across permafrost and thawing permafrost landscapes will help clarify the balance between driving mechanisms that link permafrost conditions to the LPSE and gain a quantitative understanding of environmental changes linked to the LPSE. Likewise, further studies are needed to establish whether the LPSE is observed in other regions that have experienced changing permafrost conditions, such as North America.

S1.4: Statistical investigation of potential drivers of the LPSE

S1.4.1: Bedrock geology

Bioavailable S is partly derived from the mineral weathering of parent bedrock. To assess the potential influence of geology on $\delta^{34}S$, the geological unit for each sample location was established using the USGS geological maps for Europe and Asia[40,41]. $\delta^{34}S$ values in relation to bedrock geology, as given in these maps, are shown in Figure S3 and summarised in Table S1. While $\delta^{34}S$ does vary significantly between geological category (Kruskal-Wallis chi-squared = 88.754, df = 21, p-value = 2.645e-10), post-hoc pairwise comparisons (Table S2) indicate between-group differences are only present in 7 out of 210 comparisons. Importantly, when the temporal trend in the $\delta^{34}S$ data is considered, and where sample size permits, it is apparent that the excursion to lower values occurs in areas of all rock ages and types (Figures S4 and S5).

When considering these results, it is important to recognise that the USGS continental-scale maps indicate only the age of bedrock and specify whether the rock is sedimentary or igneous. They do not provide high-resolution information on the rock type/mineral composition of each unit, which is a greater influence on $\delta^{34}S$ values than rock age. Utilising such detailed geological maps is not feasible at the spatial scale of our study. It is also important to note that in geologically diverse regions an animal's home range can span many different geologies and thus the single-point estimation is unlikely to represent the full range of geologies the animal may have lived on.

Moreover, bedrock geology has not varied over the time span of our study, thus while a comparison to geology can allow us to investigate whether a temporal change is δ^{34}S values is more likely to occur on certain rock types, bedrock geology cannot be a driver of the temporal change we observe in δ^{34}S values.

Figure S3: Faunal δ^{34}S plotted against geological category, as determined from the USGS geological maps for Europe and Asia[40,41]. Box indicates inter-quartile range (IQR), line in box indicates median, whiskers indicate 1.5 times the IQR, and points show individual data.

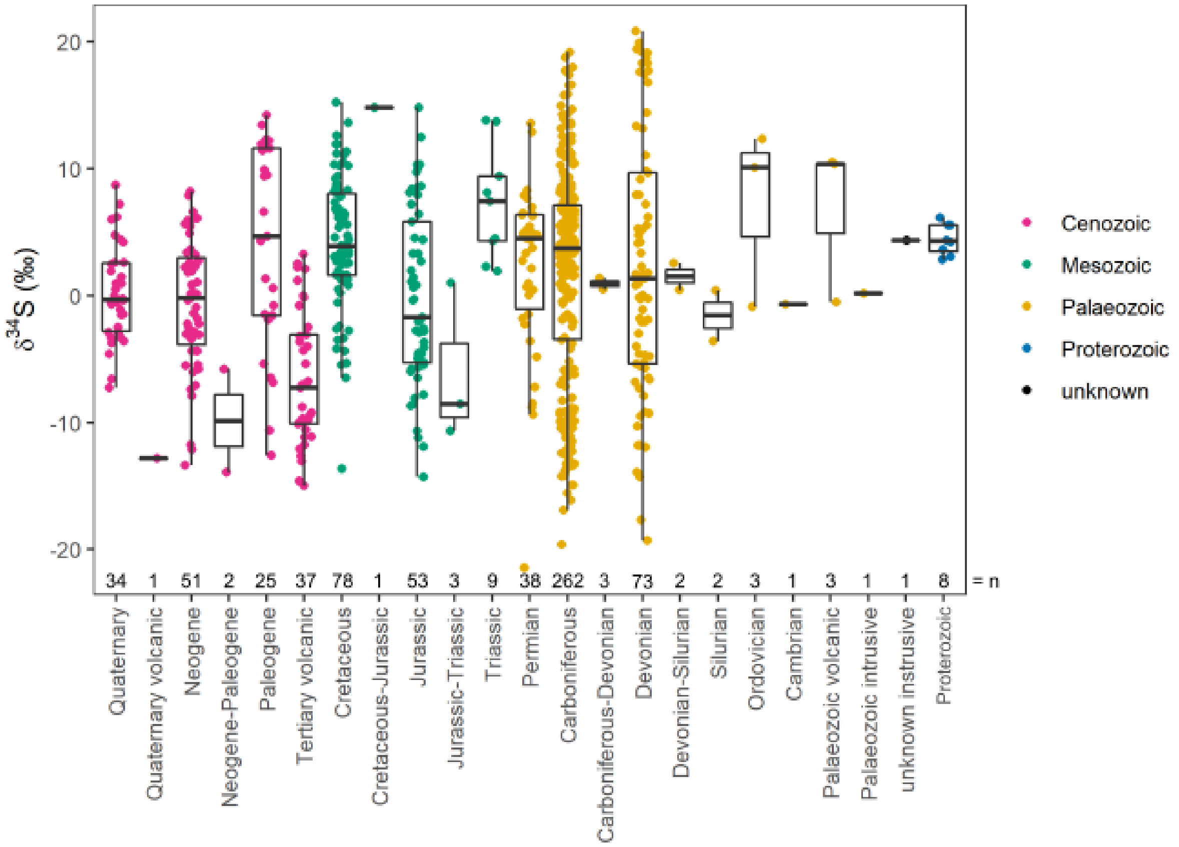

Figure S4: Faunal δ^{34}S plotted against time and colour coded according to broad geological age as determined from the USGS geological maps for Europe and Asia[40,41].

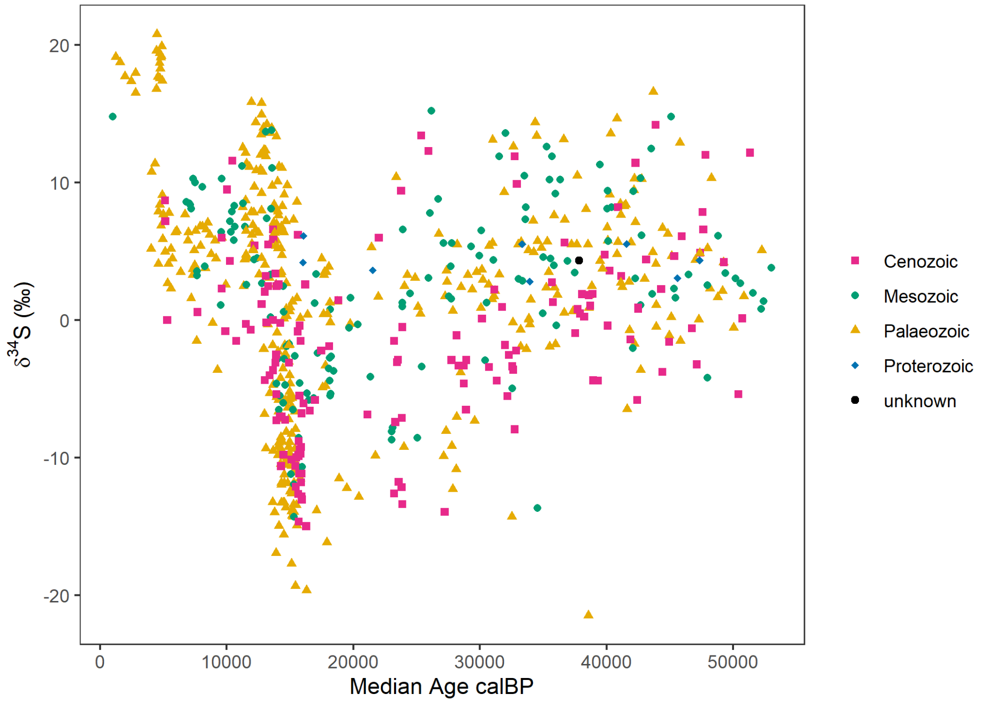

Figure S5: Faunal δ^{34}S plotted against time and colour-coded according to geological category of sedimentary and igneous rock as determined from the USGS geological maps for Europe and Asia[40,41].

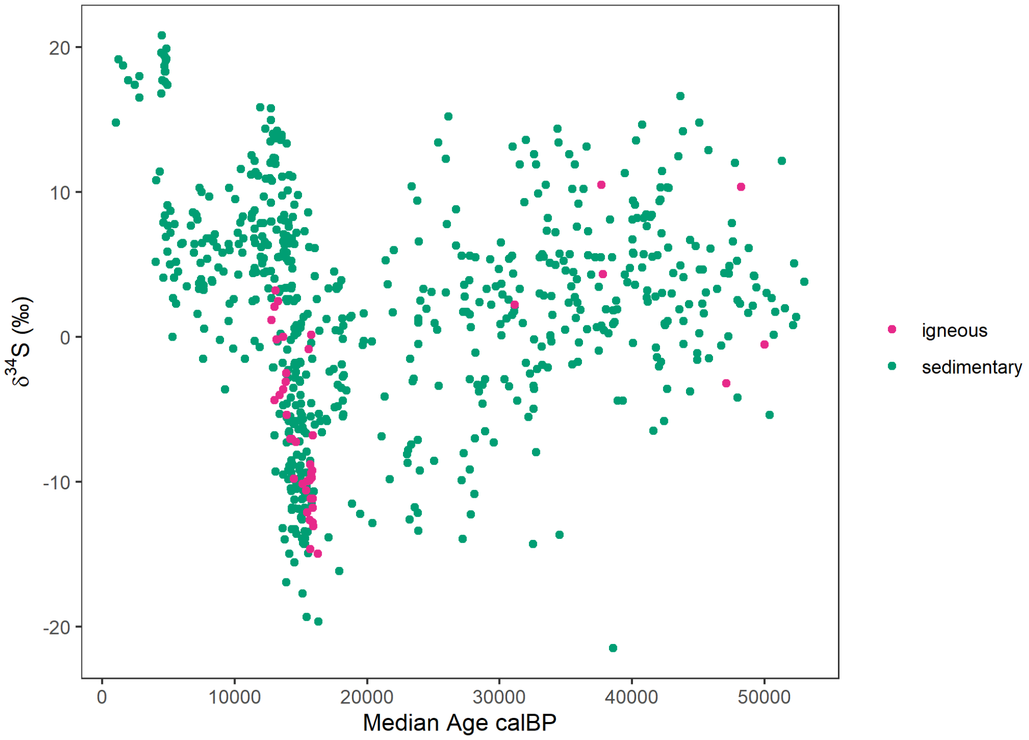

S1.4.2: Unconsolidated sediment

Unconsolidated sediments also contribute to bioavailable S. In many areas these sediments overlie bedrock and thus will likely be a bigger influencer on ecosystem $\delta^{34}S$ values. The Global Unconsolidated Sediments Map database (GUM[42,43] was used to establish unconsolidated sediment category at each sample location. Again, it is important to note that an animal's home range may span different sediment types, and thus the single-point estimation is unlikely to represent the full range of sediments the animal may have lived on. Further, erosion and deposition of unconsolidated sediments is a continual process and thus the distribution of sediments today may not precisely reflect that of the distribution at the time our animal samples acquired their $\delta^{34}S$ signature.

Unconsolidated sediment categories in relation to $\delta^{34}S$ are shown in Figure S5 and summarised in Table S3. While $\delta^{34}S$ does vary significantly between unconsolidated sediment category (Kruskal-Wallis chi-squared = 38.799, df = 14, p-value = 0.0003918), post-hoc pairwise comparisons (Table S2) indicate between-group differences are only present in 10 out of 105 comparisons. Importantly, low $\delta^{34}S$ values are present across a range of sediment types including those of aeolian, alluvial and glacial origin (Figures S5 and S6), suggesting that proximity to specific sediments is not a prerequisite/driver for temporal variation in ecosystem $\delta^{34}S$.

Figure S6: Faunal $\delta^{34}S$ plotted against unconsolidated sediment category, as determined from the Global Unconsolidated Sediments Map database (GUM)[42,43]. Box indicates inter-quartile range (IQR), line in box indicates median, whiskers indicate 1.5 times the IQR, and points show individual data.

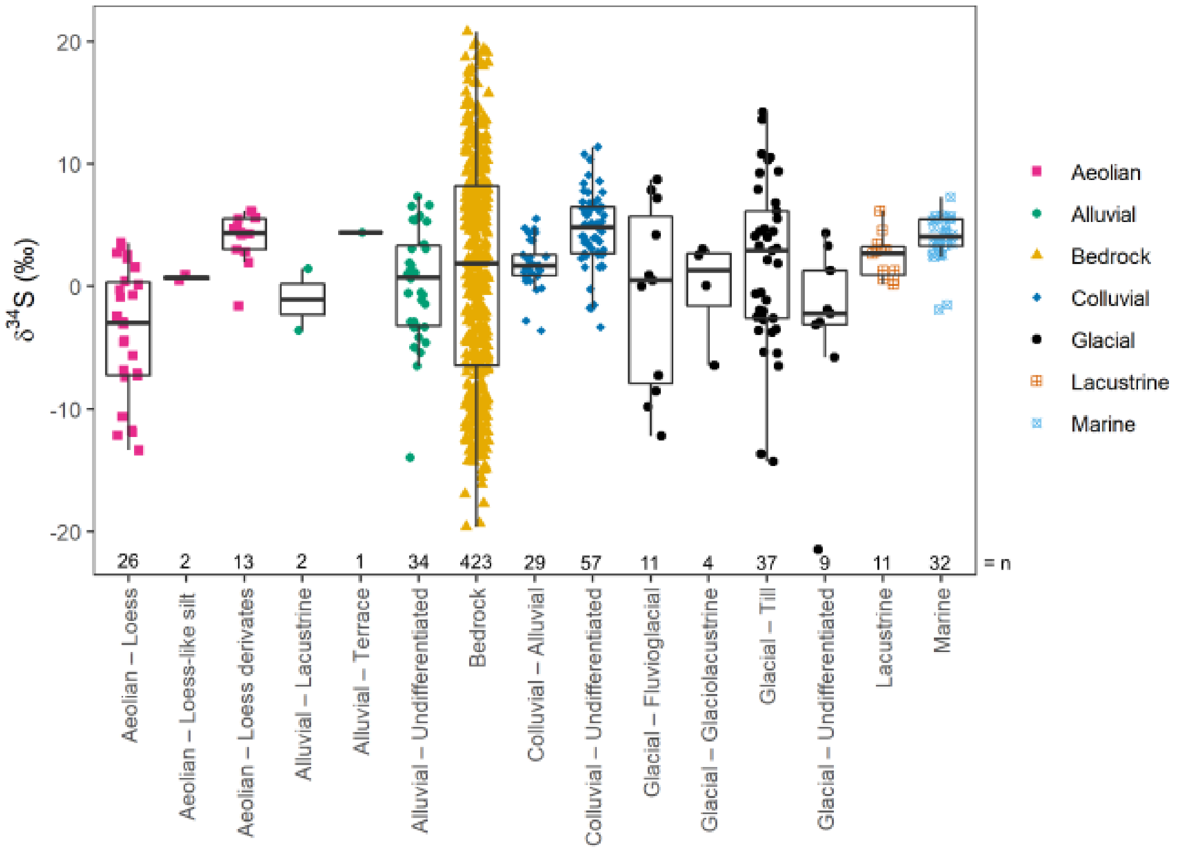

Figure S7: Faunal $\delta^{34}S$ plotted against time and colour coded according to unconsolidated sediment category as determined from the Global Unconsolidated Sediments Map database (GUM)[42,43].

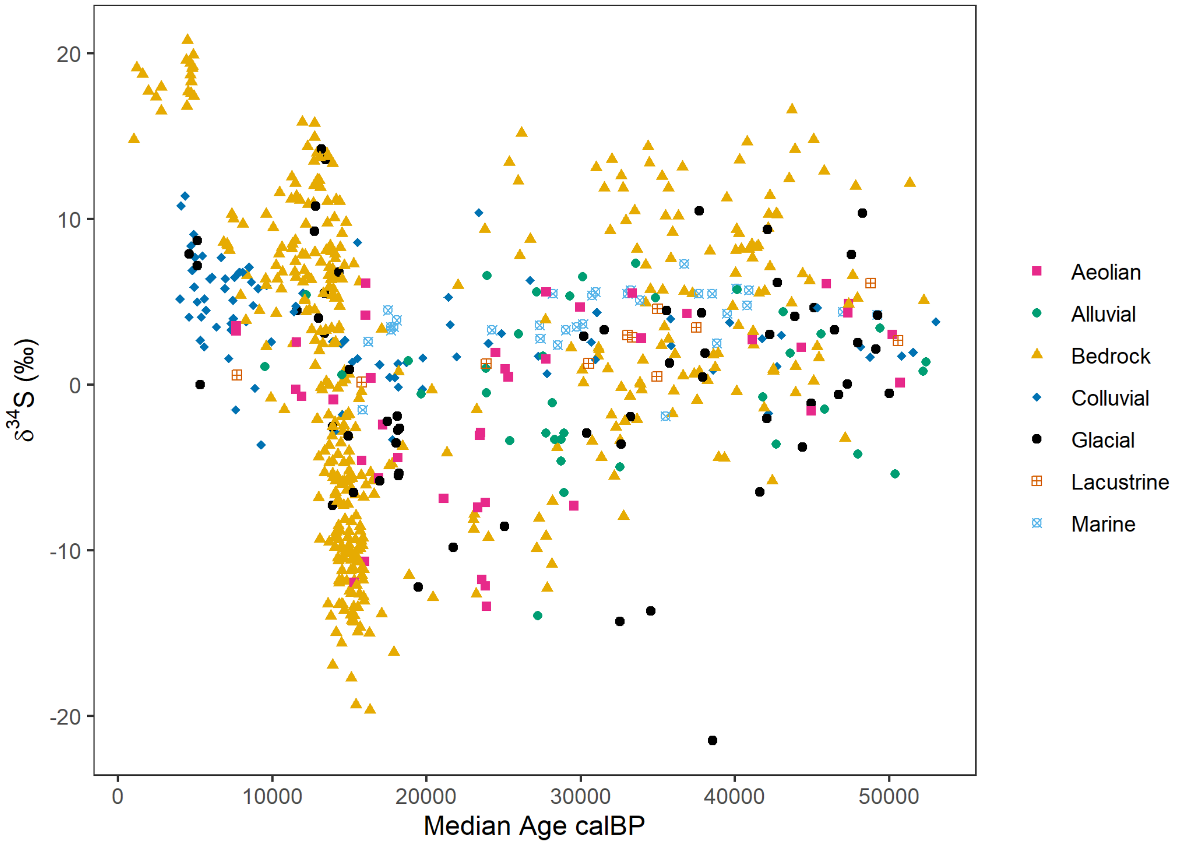

Of the unconsolidated sediment types, loess is particularly important at the spatiotemporal resolution of our study, as increased loess deposition during the last glacial was significant. Considering the distribution of loess sediments (loess, loess-like siles and loess derivates) in relation to $\delta^{34}S$, we categorise our data into 3 groups; samples with 10 km of loess sediments (i.e. animals most likely to have eaten plants grown on loessic sediments), samples within 10–50 km of loess sediments (i.e. animals with a reasonable probability of having eaten plants grown on loessic sediments), and samples more than 50 km from loess (i.e. animals least likely to have eaten plants grown on loessic sediments). The categorisation of data in this manner, rather than using absolute distance to loess, allows us to counter some of the problems of using a single-point estimation for animals, which move around the landscape. While results indicate a significant difference in $\delta^{34}S$ between samples within 10 km of loess and the other two groupings (Figure S8, Kruskal-Wallis chi-squared = 74.58, df

= 2, p-value < 2.2e-16, pairwise comparisons in Table S5), the temporal trend in $\delta^{34}S$ shows that the excursion to lower $\delta^{34}S$ during the LGM and Late Glacial occurs in both samples within 10 km of loess and those more than 50 km away from loess (Figure S9). Again, this suggests that proximity to loess sediments is not a prerequisite/driver for the temporal variation we observed in the faunal $\delta^{34}S$ data.

Figure S8: Faunal $\delta^{34}S$ plotted against loess sediment categories, as determined from the Global Unconsolidated Sediments Map database (GUM)[42,43]. Box indicates inter-quartile range (IQR), line in box indicates median, whiskers indicate 1.5 times the IQR, and points show individual data.

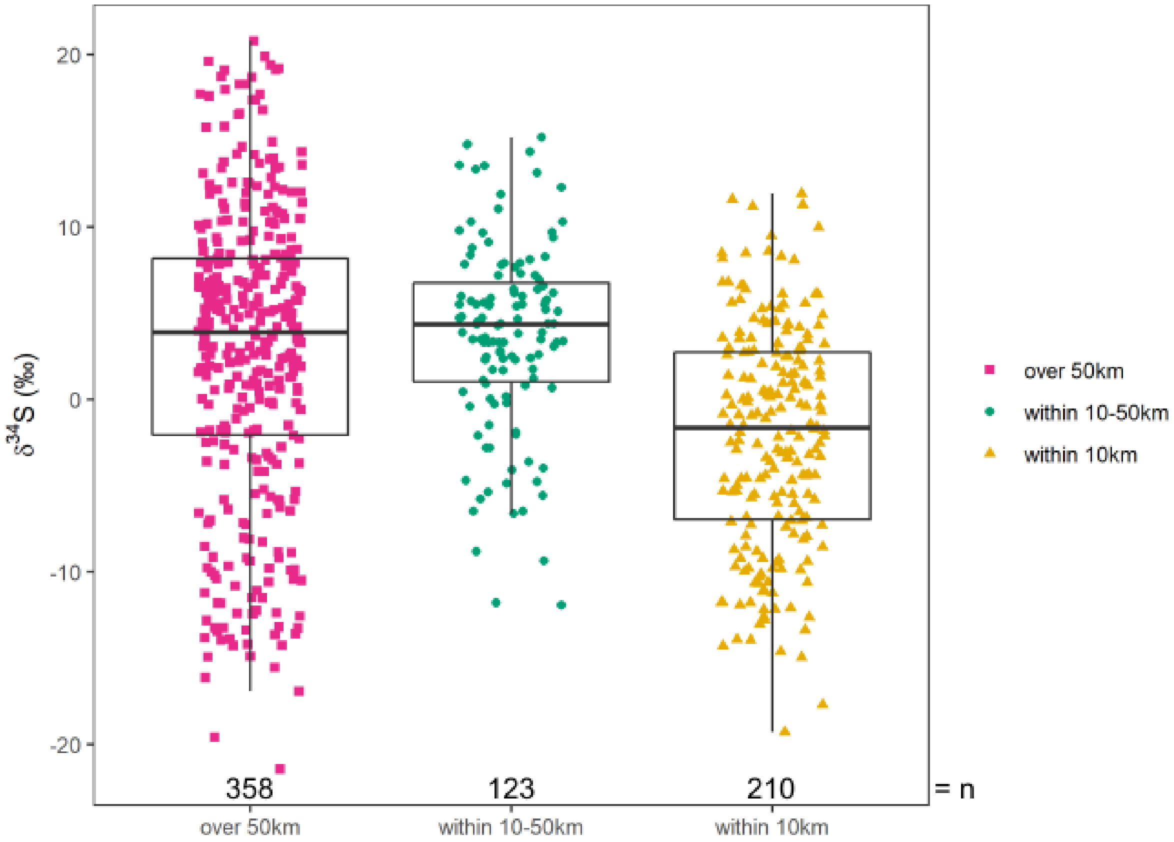

Figure S9: Faunal $\delta^{34}S$ plotted against time and colour coded according to distance to loess sediment category.

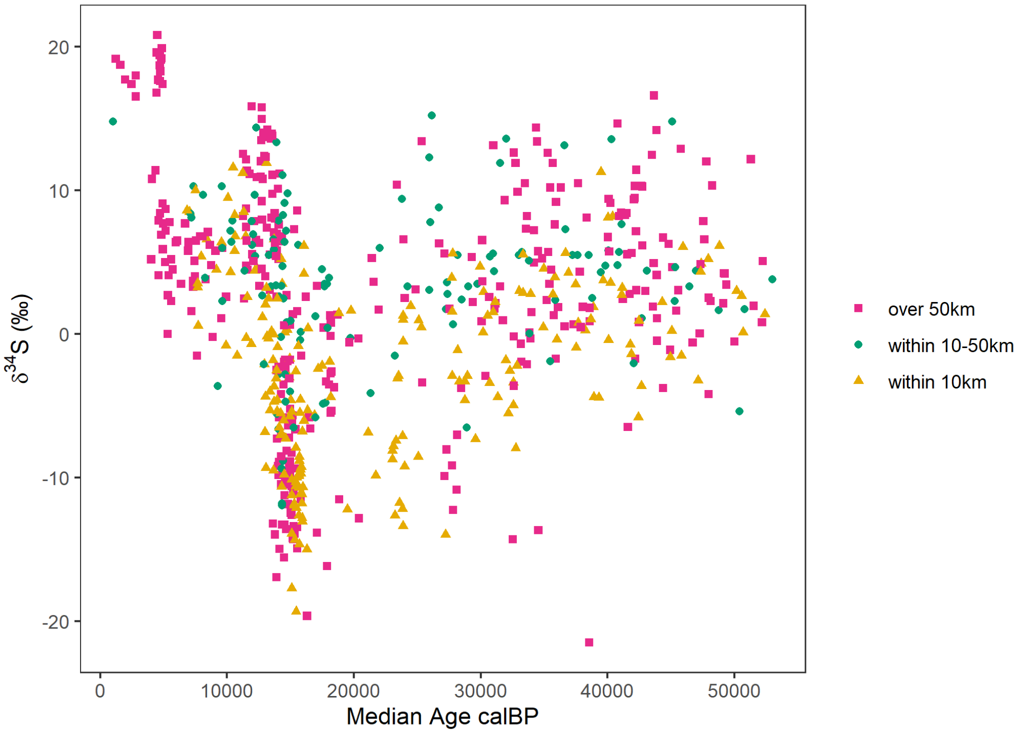

S1.4.3: Palaeoclimate, hydrosphere and cryosphere

Sea level, ice cover, and temperature all varied considerably over the time span of our study. Input of sulfur from marine and glacial sources can influence bioavailable $\delta^{34}S$ in locations proximal to these sources, while climate change can alter the biogeochemical cycling of S in the soil environment. For these variables we accessed spatially-resolved data at appropriate temporal resolutions (1,000 to 2,500 year time slices) to enable consideration against spatiotemporal variability in $\delta^{34}S$.

Mean annual air temperature and precipitation were established for each sample using Beyer's model[24], which provides 0.5° resolution bias-corrected gridded reconstructions of MAAT in 1,000-year time steps from 0 to 21,000 BP and 2000 year time steps from 22,000 to 120,000 BP. For each sample, the median calibrated BP age estimate of its radiocarbon date was rounded to the nearest 1,000 years (for samples <22,000 years cal BP) or nearest 2000 years (for samples >22,000 years cal BP), and temperature was extracted from the corresponding Beyer model[24] time step for the sample location. Where samples produced NA values from the raster due to

differences in grid resolution, the nearest cell to return a temperature/precipitation value that was not NA was used (this was only the case for Links of Noltland, Westray sites in Orkney).

Proximity of each sample (in kilometres) to the coast was established using the palaeotopographic models from PaleoMIST 1.0, which provides 2,500 year time-step data at 5 km resolution[44]. For each samples, the median calibrated BP age estimate was rounded to the nearest 2500 years and distance to the corresponding time-matched shoreline was measured. Similarly, proximity of each sample (in kilometres) to ice-sheet margins was established using the DATED-1 Eurasian Ice Sheet database[45,46]. The DATED-1 database provides ice-sheet reconstructions at 1000-year time intervals from 10,000 to 25,000 years BP. For these time steps we used the 'most credible' reconstruction from the database[45,46]. The DATED-1 database also provides ice sheet reconstructions for 4 further time intervals: 27, 29–28, 32–30, and 38–35 ka. For the first 3 of these a single extent is available. For the 38–35 ka time step a maximum and minimum likely ice extent is available; for this time slice the distance from both the max and min were measured and an average (mid-point) was taken. Where our samples, with their median cal BP ages rounded to the nearest 1000-years, did not correspond to a time interval in the DATED-1 database, no time-matched distance was calculated.

Considering these spatiotemporal factors independently, it can be seen that low $\delta^{34}S$ values (<–10 per mil) occur in environments predominately where MAAT is between around 0 and 7ºC (Figure S10 (a)) and mean annual precipitation is between around 500 and 1250 mm/year (Figure S10 (b)). Likewise, low $\delta^{34}S$ values appear to only occur within around 1200 km of ice sheets (Figure S10 (c)), while the highest $\delta^{34}S$ values (>15 per mil) appear to only occur within 250 km of the coast (Figure S10 (d)). However, it is important to recognise that while extremely high or low values appear to be restricted to certain geographical settings, these geographical settings do not exclusively produce values at these range extremes. For example, $\delta^{34}S$ values within 250 km of the coast range from –15 to +20 per mil. Crucially, this highlights the fact that it is unlikely that a sole factor is responsible for the observed geographic differences in the $\delta^{34}S$ temporal trend.

Fig S10. Faunal $\delta^{34}S$ plotted against (a) mean annual air temperature, (b) mean annual precipitation, (c) distance from ice sheet, and (d) distance for palaeo-coastline as determined from the palaeotopographic models from PaleoMIST 1.0, the DATED-1 Eurasian Ice Sheet database, and the Beyer et al. palaeoclimate model[24,44-46].

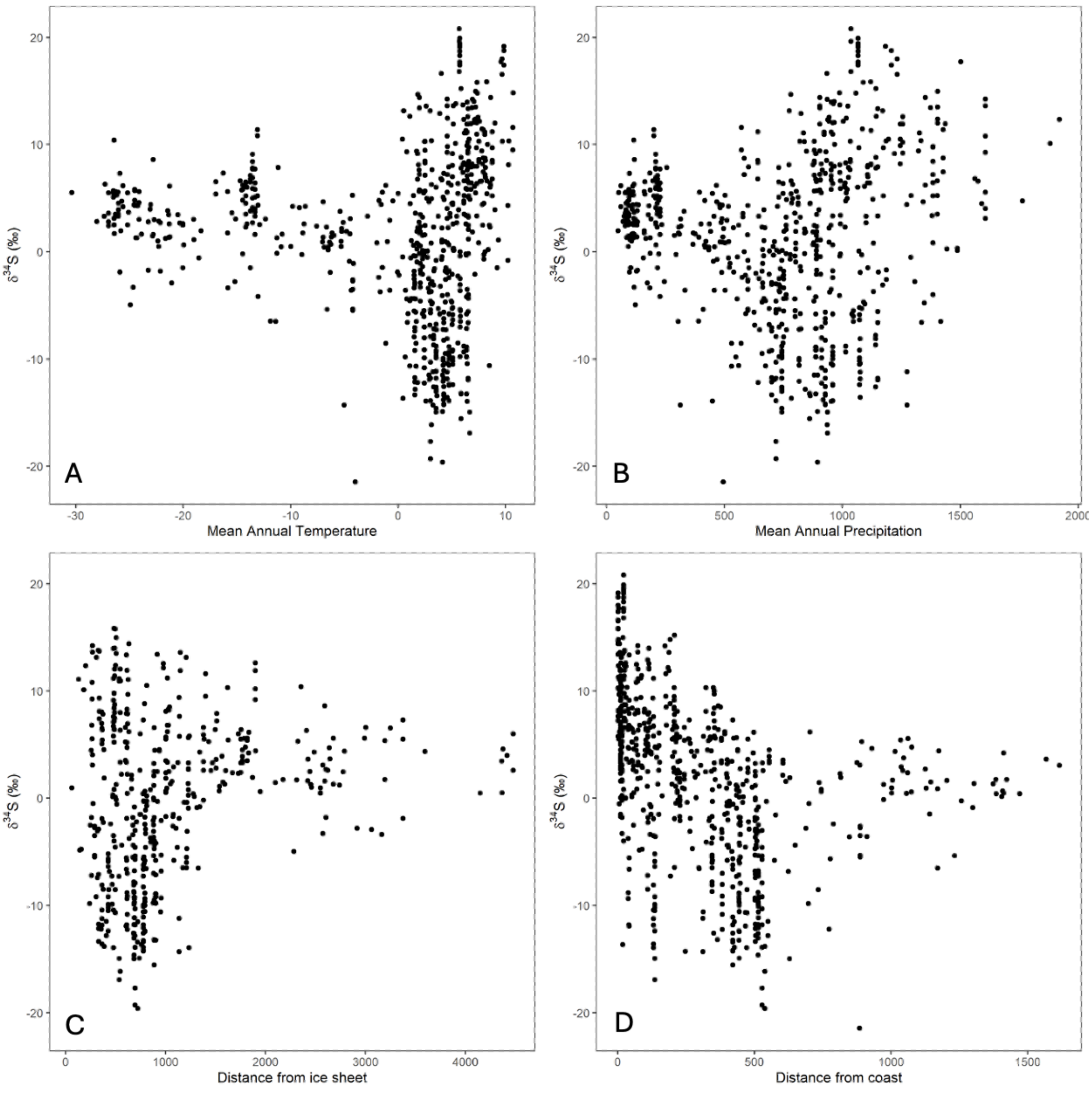

S1.4.4: Permafrost

The distribution of permafrost has also varied very significantly across the time and geographical span of our study. For a sample's proximity to permafrost to be reliably determined, permafrost distribution maps covering the last 50,000 years at time slices of 1–2 ka are needed, yet no such data are available. Instead, we can establish the proximity of each sample to present day[47] and LGM permafrost distributions[48]. Using present-day permafrost distribution map[47] we categorise our data as either being on permafrost (either continuous or discontinuous) or not. From this data categorisation we found thirty-five samples which plotted outside of, but less than 10 km away from, these zones. On further examination, 32 of these samples were found to be located on the arctic coast or arctic islands, and their plotting outside of the zone of permafrost occurred due to discrepancies between the resolution of the permafrost distribution map and the precision of the sample's coordinates. These samples were therefore included as being within the zone of modern permafrost. It could not be determined with certainty whether the three remaining samples (from the Lake Baikal area and northwest Mongolia) were from within the zone of modern continuous/discontinuous permafrost as these retain their original classification as being from outside of it. Using the LGM permafrost distribution map[48] we categorise our data as being within/outside of the LGM permafrost zone if they plotted within/outside of the boundary of the distribution of continuous LGM permafrost, respectively. An exception to this was for samples that were located under glacial ice at the LGM. For these, samples that come from locations north of the southern latitudinal limit of the zone of continuous LGM permafrost are considered as being from within the zone of continuous LGM permafrost, while samples that come from locations south of the southern latitudinal limit of the zone of continuous LGM permafrost are considered as being from outside the zone of continuous LGM permafrost. Our rationale for this is that areas subjected to glaciation at the LGM that are within the latitudinal zone of continuous LGM permafrost most likely were also subjected to widespread permafrost development either immediately before and/or after glaciation, while this is less likely for glaciated areas outside of the latitudinal zone of continuous LGM permafrost. Combining the categories for present-day and LGM permafrost, allowed us to incorporate a variable for permafrost that included a time dimension; each sample was assigned to one of

three classifications; “PF present throughout”, “PF absent throughout”, or “no PF today, PF at LGM”.

Permafrost categories in relation to $\delta^{34}S$ are shown in Figure S11 and summarised in Table S6. Faunal $\delta^{34}S$ varies significantly between permafrost categories (Kruskal-Wallis chi-squared = 64.937, df = 2, p-value = 7.928e-15, pairwise comparisons in Table S7) and the temporal trend in $\delta^{34}S$ clearly shows that the excursion to lower $\delta^{34}S$ occurs only in samples assigned to the “no PF today, PF at LGM” category (Figure S12). These results suggest there is a strong link between permafrost development and thaw and the variation we observe in the faunal $\delta^{34}S$ data.

Figure S11: Faunal $\delta^{34}S$ plotted against permafrost categories as determined from present day[47] and LGM permafrost[48] distribution maps. Box indicates inter-quartile range (IQR), line in box indicates median, whiskers indicate 1.5 times the IQR, and points show individual data.

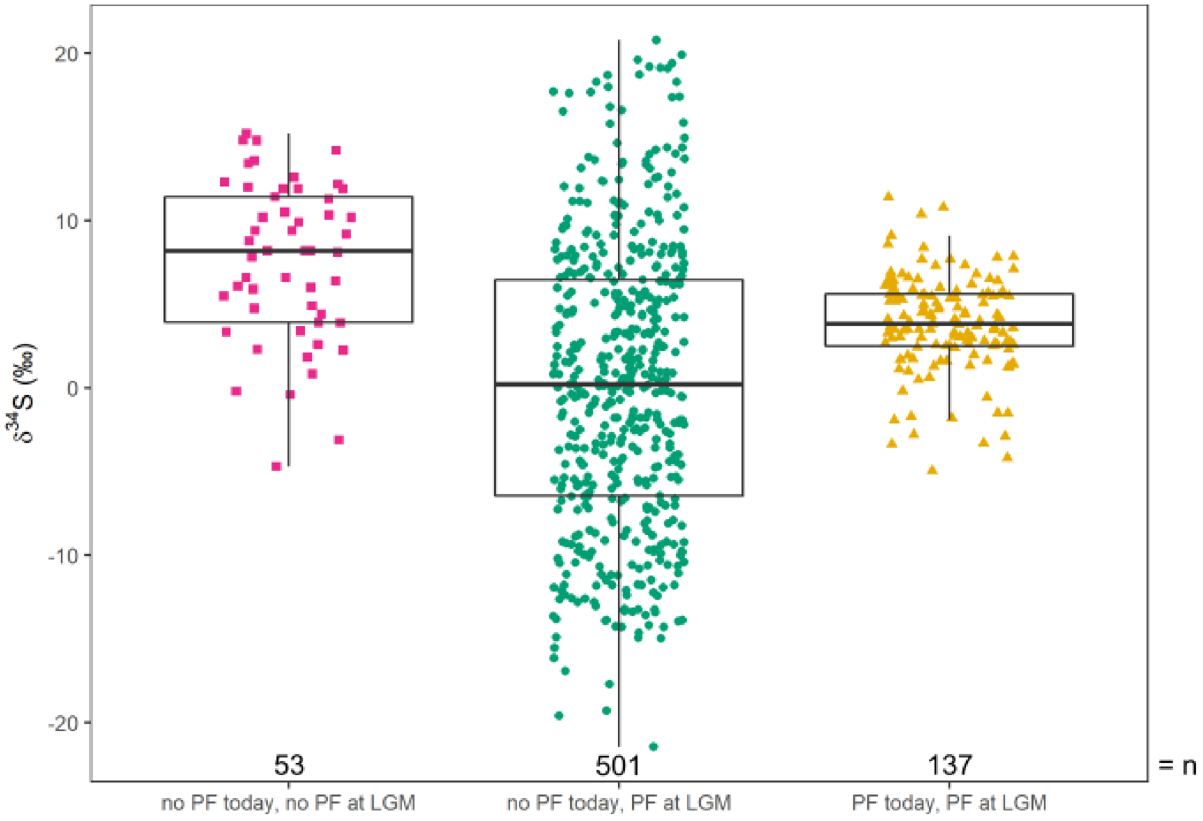

Figure S12: Faunal $\delta^{34}S$ plotted against time and colour coded according to permafrost category.

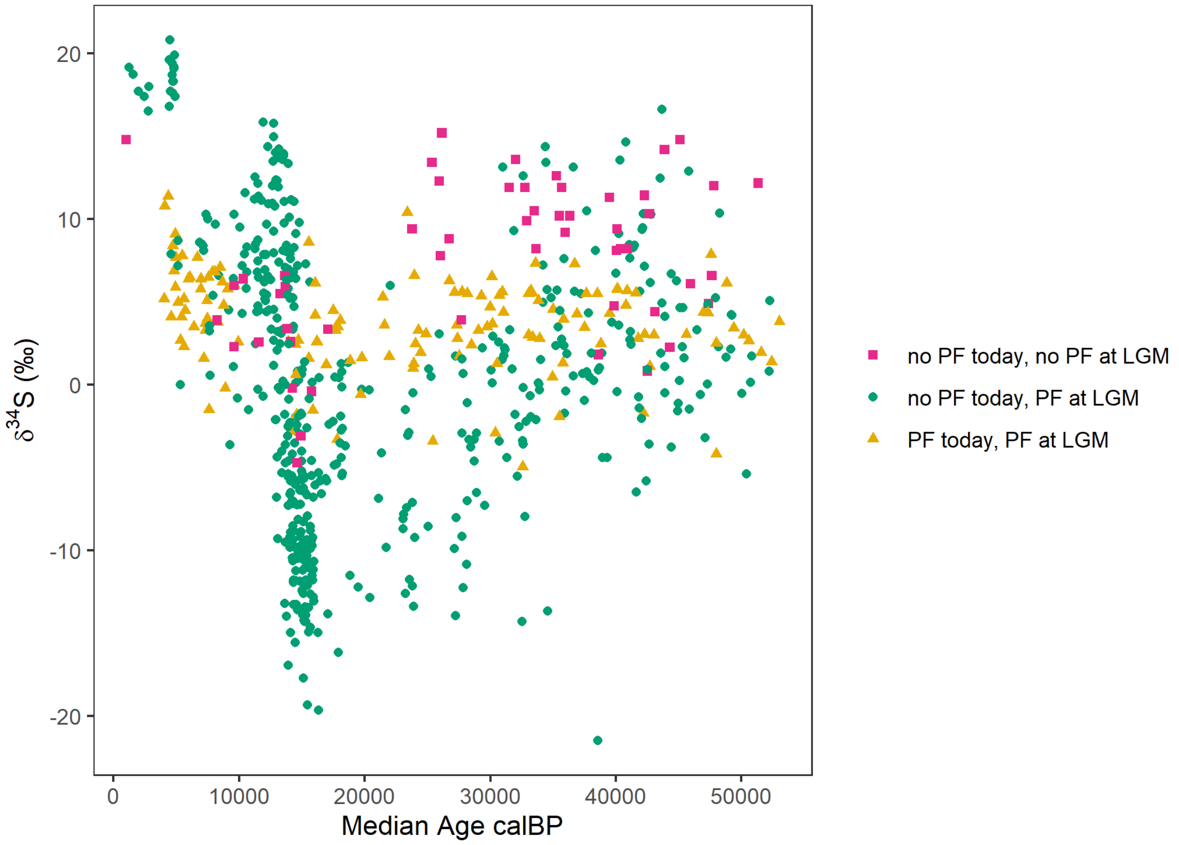

S1.4.5: Multivariate Analysis

Our multivariate statistical analysis of our data (FAMD analysis and hierarchical cluster analysis) shows that while there is not a sole factor responsible for the observed geographic differences in the $\delta^{34}S$ temporal trend, permafrost history (i.e., present at the LGM but not today, present throughout the last 50 kyr, or absent during last 50 kyr) is by far the most important factor. MAAT and precipitation, and distance to ice-sheet margins are influencing variables, but permafrost history best explains the observed geographic differences in the $\delta^{34}S$ values, followed by proximity to loess (Fig. S13, Fig. S14, Table S8, S9). Although proximity to loess does influence faunal $\delta^{34}S$ values, the LPSE is present both in data from regions proximal to and distant from loess deposits; thus loess is not driving the LPSE. Overall, our multivariate analysis (Fig. S15, Fig. S16, Fig. S17) demonstrates a clear relationship between the

temporal variation in faunal $\delta^{34}S$ values and permafrost history, suggesting that changing permafrost conditions is the main driver of the LPSE.

It is also clear that many of the potential influencing variables considered here are not independent from one another. For example, the closer a sample is to the ice-sheet margin, the colder the air temperature is likely to be, and the more likely it is that permafrost will be present. To enable the potential drivers of $\delta^{34}S$ variation to be considered together a factor analysis of mixed data (FAMD) approach was used to reduce the dimensionality of the data. FAMD is a multivariate principal component method which enables continuous and categorical variables to be considered within the same model.

FAMD analysis was conducted using the R packages "FactoMineR” and "factoextra". The first three principal components (eigenvalues >1) were found to explain 76% of the variance (Figure S13, Table S8). The percentage contribution of each variable to each of these three dimensions, and their combined contribution across the three dimensions is shown in Figure S14 and Table S9. This shows, for the first dimension (which explains 42.1% of the variance), permafrost category, MAAT and precipitation, and distance to ice-sheet margins are the most important variables (Table S9). Across all three dimensions, the permafrost category contributed the most, followed by the loess category (Figure S14).

Figure S13: Screeplot showing the dimensions identified in the FAMD analysis and their corresponding percentage of explained variance.

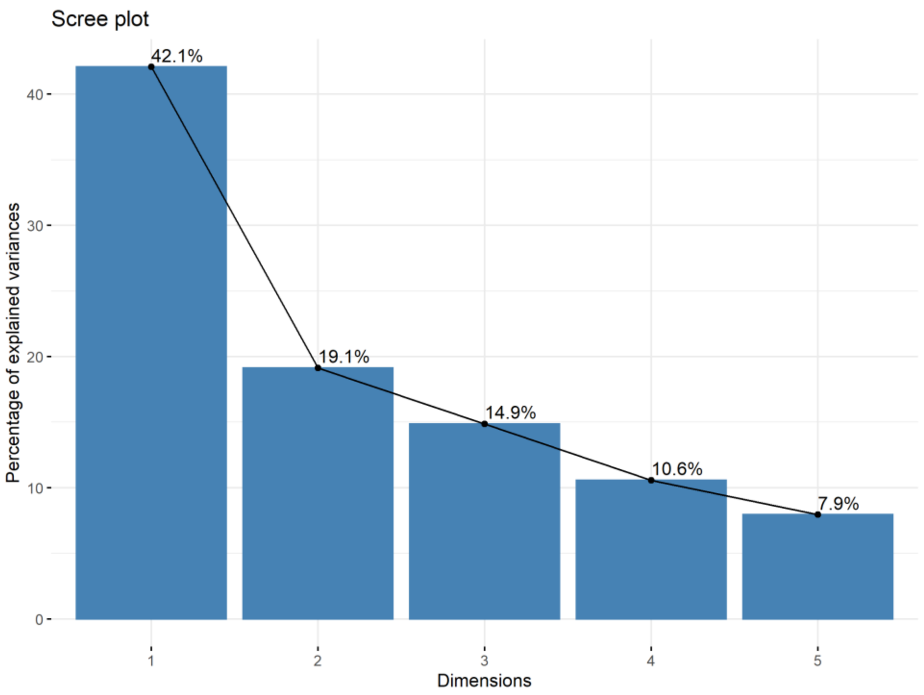

Figure S14: Plots showing the percentage contributions of each variable to each dimension identified in the FAMD analysis. The red dashed line on the graphs above indicates the expected average value, if each variable's contribution was uniform.

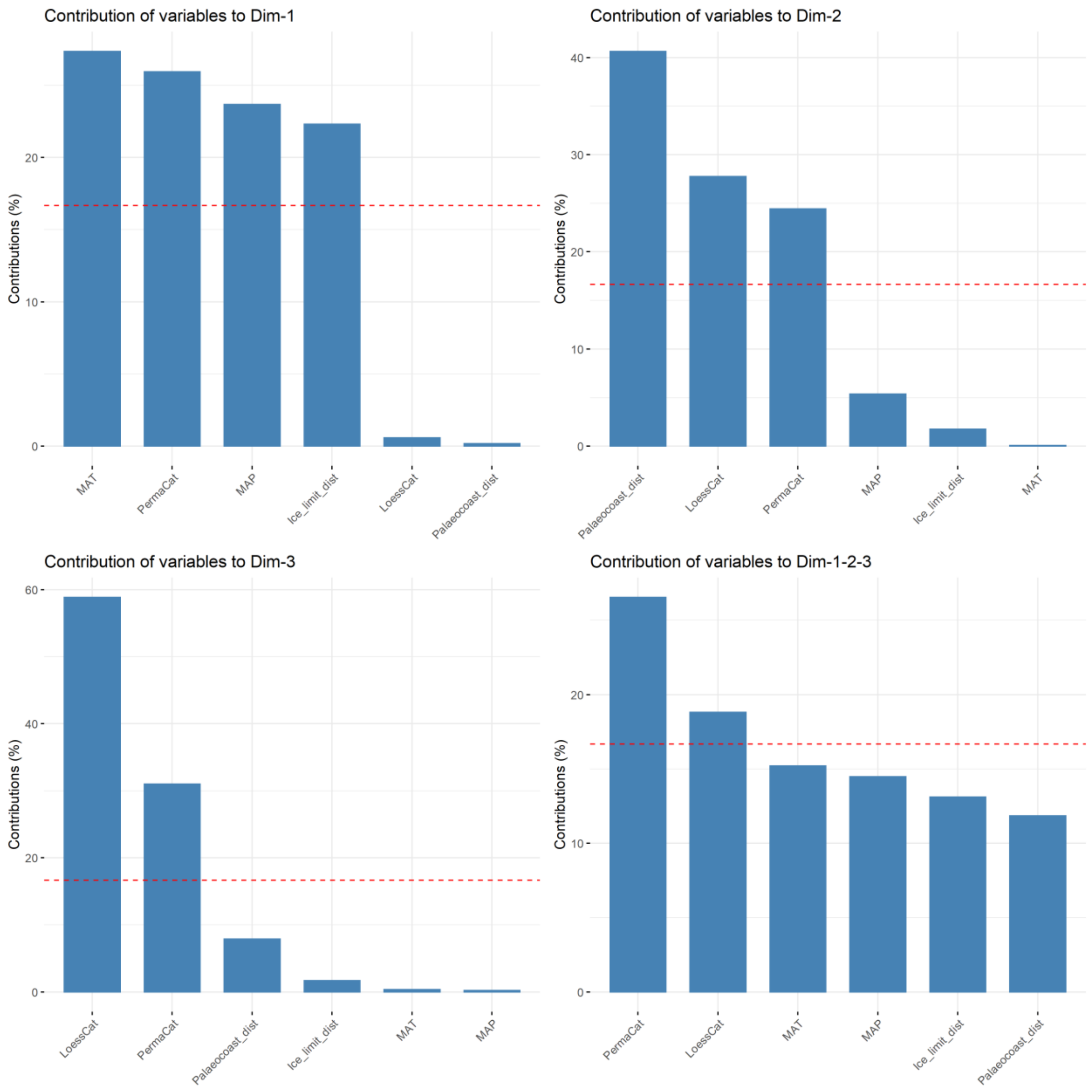

A hierarchical cluster analysis using the three most important components obtained from the FAMD analysis was then conducted (Figure S15). This method identifies subgroups in the data which share similar characteristics, providing a means to explore which variables may be contributing to the observed $\delta^{34}S$ trends. From this analysis (Figure S16), it can be shown that cluster 1 is very strongly separated from the other clusters based on the permafrost category 'PF today, PF at LGM', and where MAAT is <–10ºC, and MAP is <250 mm/year. Clusters 2 and 4 are both dominated by

data from the permafrost category ‘no PF today, PF at LGM’, but separate strongly from one another based on the proximity to loess category (‘within 10 km’ for Cluster 2 and ‘over 50 km’ for Cluster 4). Cluster 3 contains data from both ‘no PF today, PF at LGM’ and ‘no PF today, no PF at LGM’ categories and notably is the only cluster to contain data where MAAT estimates are exclusively above 0ºC.

Considering these clusters in relation to the temporal trend observed in the $\delta^{34}S$ data (Figure S17) a clear pattern emerges; the LPSE is very clearly expressed in both clusters 2 and 4, suggesting that permafrost category is more important in the $\delta^{34}S$ signature, while proximity to loess is not. The LPSE is absent in Cluster 1 and largely absent in Cluster 3, bar a few low values (<–5 per mil) in the Late Glacial. Interrogation of these low $\delta^{34}S$ data points show these all fall within the ‘no PF today, PF at LGM’ category. This analysis demonstrates a clear temporal correlation between $\delta^{34}S$ and permafrost.

Figure S15: Clusters plotted by principal component dimensions.

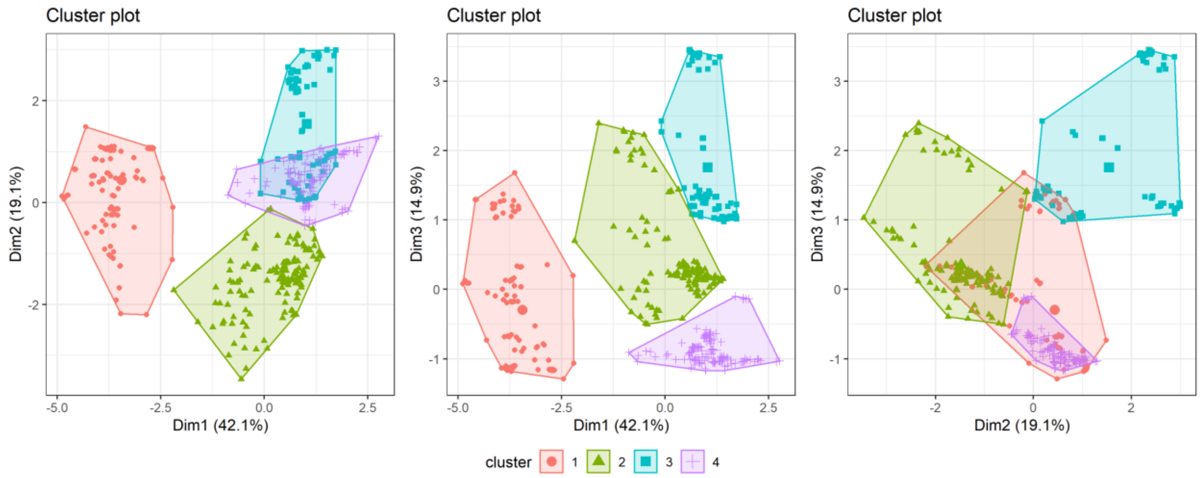

Figure S16 (next page): Data distribution by cluster plotted for each variable considered in the FAMD and hierarchical cluster analysis.

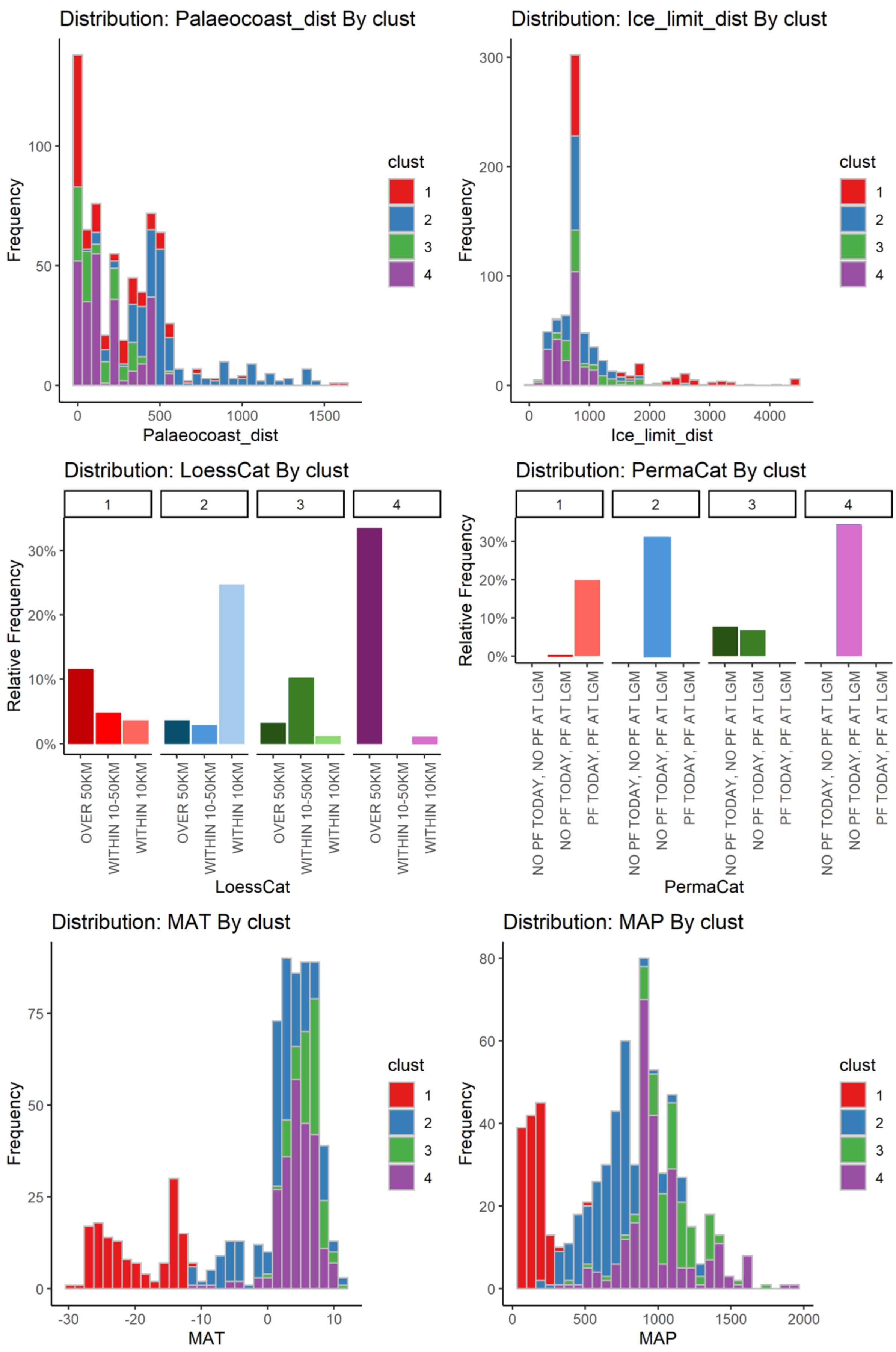
Distribution: Palaeocoast_dist By clust
Frequency
Palaeocoast_dist
0
50
100
500
1000
1500
clust
1
2
3
4
Distribution: Ice_limit_dist By clust
Frequency
Ice_limit_dist
0
100
200
300
1000
2000
3000
4000
clust
1
2
3
4
Distribution: LoessCat By clust
Relative Frequency
LoessCat
0%
10%
20%
30%
OVER 50KM
WITHIN 10-50KM
WITHIN 10KM
Distribution: PermaCat By clust
Relative Frequency
PermaCat
0%
10%
20%
30%
NO PF TODAY, NO PF AT LGM
NO PF TODAY, PF AT LGM
PF TODAY, PF AT LGM
Distribution: MAT By clust
Frequency
MAT
0
25
50
75
-30
-20
-10
10
clust
1
2
3
4
Distribution: MAP By clust
Frequency
MAP
0
20
40
60
80
500
1000
1500
2000
clust
1
2
3
4

Figure S17: Faunal $\delta^{34}S$ plotted through time, colour coded by assigned cluster.

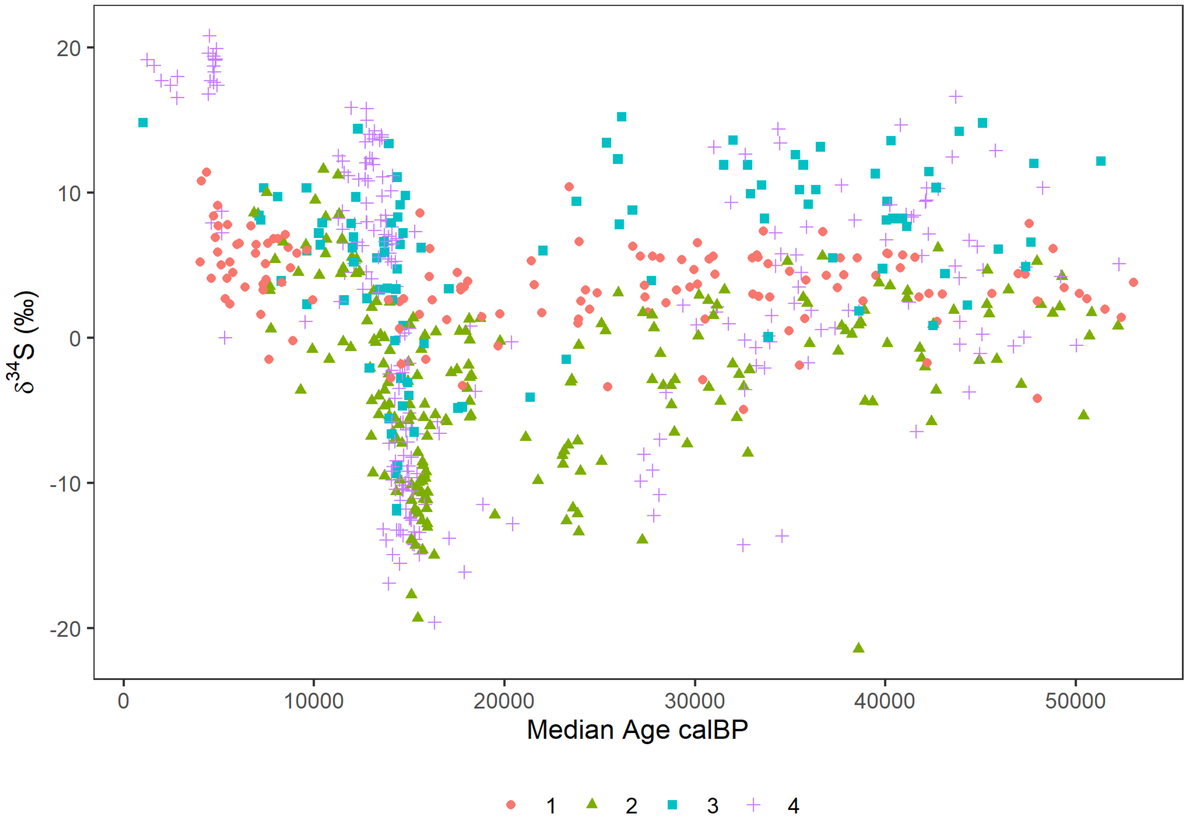

References:

1. North Greenland Ice Core Project members. High-resolution record of Northern Hemisphere climate extending into the last interglacial period. *Nature* **431**, 147–151 (2004).
2. Bertran, P. Distribution and characteristics of Pleistocene ground thermal contraction polygons in Europe from satellite images. *Permafrost Periglacial Processes* **33**, 99–113 (2022).
3. Wolff, E. W., Chappellaz, J., Blunier, T., Rasmussen, S. O. & Svensson, A. Millennial-scale variability during the last glacial: The ice core record. *Quat. Sci. Rev.* **29**, 2828–2838 (2010).
4. Nehlich, O. The application of sulphur isotope analyses in archaeological research: A review. *Earth-Science Reviews* vol. 142 1–17 (2015).
5. Zazzo, A., Monahan, F. J., Moloney, A. P., Green, S. & Schmidt, O. Sulphur isotopes in animal hair track distance to sea. *Rapid Commun. Mass Spectrom.* **25**, 2371–2378 (2011).
6. Bataille, C. P. *et al.* Triple sulfur-oxygen-strontium isotopes probabilistic geographic assignment of archaeological remains using a novel sulfur isoscape of western Europe. *PLoS One* **16**, e0250383 (2021).

7. Lambeck, K., Yokoyama, Y. & Purcell, T. Into and out of the Last Glacial Maximum: sea-level change during Oxygen Isotope Stages 3 and 2. *Quat. Sci. Rev.* **21**, 343–360 (2002).
8. Lambeck, K., Rouby, H., Purcell, A., Sun, Y. & Sambridge, M. Sea level and global ice volumes from the Last Glacial Maximum to the Holocene. *Proc. Natl. Acad. Sci. U. S. A.* **111**, 15296–15303 (2014).
9. Ballin, T. B. Rising waters and processes of diversification and unification in material culture: the flooding of Doggerland and its effect on north-west European prehistoric populations between ca. 13 000 and 1500 cal BC. *J. Quat. Sci.* **32**, 329–339 (2017).
10. Uemura, R. *et al.* Soluble salts in deserts as a source of sulfate aerosols in an Antarctic ice core during the last glacial period. *Earth Planet. Sci. Lett.* **578**, 117299 (2022).
11. Lehmkuhl, F. *et al.* Loess landscapes of Europe – Mapping, geomorphology, and zonal differentiation. *Earth-Sci. Rev.* **215**, 103496 (2021).
12. Shennan, I. & Andrews, J. An introduction to Holocene land-ocean interaction and environmental change around the western North Sea. *Geological Society, London, Special Publications* **166**, 1–7 (2000).
13. Ward, I., Larcombe, P. & Lillie, M. The dating of Doggerland – post-glacial geochronology of the southern North Sea. *Environmental Archaeology*. **11**, 207–218 (2006).
14. Burke, A. *et al.* Stratospheric eruptions from tropical and extra-tropical volcanoes constrained using high-resolution sulfur isotopes in ice cores. *Earth Planet. Sci. Lett.* **521**, 113–119 (2019).
15. Robock, A. Volcanic eruptions and climate. *Rev. Geophys.* **38**(2), 191-219 (2000).
16. Lin, J. *et al.* Magnitude, frequency and climate forcing of global volcanism during the last glacial period as seen in Greenland and Antarctic ice cores (60–9 ka). *Clim. Past.* **18**, 485–506 (2022).
17. Thode, H. G. Sulphur isotopes in nature and the environment: an overview. *Stable isotopes: natural and anthropogenic sulphur in the environment.* **43**, 1–26 (1991).
18. Tuffen, H. How will melting of ice affect volcanic hazards in the twenty-first century? *Philos. Trans. A Math. Phys. Eng. Sci.* **368**, 2535–2558 (2010).
19. Lin, J. *et al.* Magnitude, frequency and climate forcing of global volcanism during the last glacial period as seen in Greenland and Antarctic ice cores (60–9 ka). *Clim. Past* **18**, 485–506 (2022).
20. Baldini, J., Brown, R. J. & Mawdsley, N. Evaluating the link between the sulfur-rich Laacher See volcanic eruption and the Younger Dryas climate anomaly. *Clim. Past.* **14**(7), 969-990 (2018)
21. Riede, F. Changes in mid- and far-field human landscape use following the Laacher See eruption (c. 13,000 BP). *Quat. Int.* **394**, 37–50 (2016).
22. Patton, H. *et al.* Deglaciation of the Eurasian ice sheet complex. *Quat. Sci. Rev.* **169**, 148–172 (2017).

23. Bartlein, P. J. *et al.* Pollen-based continental climate reconstructions at 6 and 21 ka: a global synthesis. *Clim. Dyn.* **37**, 775–802 (2011).
24. Beyer, R. M., Krapp, M. & Manica, A. High-resolution terrestrial climate, bioclimate and vegetation for the last 120,000 years. *Sci Data* **7**, 236 (2020).
25. Zolkos, S., Tank, S. E. & Kokelj, S. V. Mineral weathering and the permafrost carbon-climate feedback. *Geophys. Res. Lett.* **45**, 9623–9632 (2018).
26. Postgate J. Sulphate reduction by bacteria. *Annual Reviews in Microbiology* **13**(1),505–520 (1959).
27. Kemp, A. L. W. & Thode, H. G. The mechanism of the bacterial reduction of sulphate and of sulphite from isotope fractionation studies. *Geochim. Cosmochim. Acta* **32**, 71–91 (1968).
28. Chambers, L. A. & Trudinger, P. A. Microbiological fractionation of stable sulfur isotopes: A review and critique. *Geomicrobiol. J.* **1**, 249–293 (1979).
29. Toran, L. & Harris, R. F. Interpretation of sulfur and oxygen isotopes in biological and abiological sulfide oxidation. *Geochim. Cosmochim. Acta* **53**, 2341–2348 (1989).
30. Krouse, R., Mayer, B., & Schoenau, J. J. Applications of stable isotope techniques to soil sulfur cycling. in *Mass Spectrometry of Soils* (eds. Boutton, T. & Yamasaki, S.) 247–284 (Marcel Dekker Inc, New York) (1996).
31. Lamers, L. *et al.* Sulfide as a soil phytotoxin—a review. *Front. Plant Sci.* **4**, 268 (2013).
32. Van Stempvoort, D. & Biggar, K. Potential for bioremediation of petroleum hydrocarbons in groundwater under cold climate conditions: A review. *Cold Reg. Sci. Technol.* **53**, 16–41 (2008).
33. Van Stempvoort, D. R. *et al.* Sulfate in streams and groundwater in a cold region (Yukon Territory, Canada): Evidence of weathering processes in a changing climate. *Chem. Geol.* **631**, 121510 (2023).
34. Ansari, A. H. Stable isotopic evidence for anaerobic maintained sulphate discharge in a polythermal glacier. *Polar Sci.* **10**, 24–35 (2016).
35. Hindshaw, R. S., Heaton, T. H. E., Boyd, E. S., Lindsay, M. R. & Tipper, E. T. Influence of glaciation on mechanisms of mineral weathering in two high Arctic catchments. *Chem. Geol.* **420**, 37–50 (2016).
36. Jones, E. L. *et al.* Biogeochemical Processes in the Active Layer and Permafrost of a High Arctic Fjord Valley. *Front. Earth Sci.* **8**, 342 (2020).
37. Kemeny, P. C. *et al.* Arctic permafrost thawing enhances sulfide oxidation. *Global Biogeochem. Cycles* **37**, (2023).
38. French, H. & Shur, Y. The principles of cryostratigraphy. *Earth-Sci. Rev.* **101**, 190–206 (2010).
39. Mitchell, K., Heyer, A., Canfield, D. E., Hoek, J. & Habicht, K. S. Temperature effect on the sulfur isotope fractionation during sulfate reduction by two strains of the hyperthermophilic Archaeoglobus fulgidus. *Environ. Microbiol.* **11**, 2998–3006 (2009).

40. Persits, F.M., Ulmishek, G.F. & Steinshouer, D.W. Maps showing geology, oil and gas fields and geologic provinces of the former Soviet Union: *U.S. Geological Survey Open-File Report* **97**(470-E), 13 p. (1999).
41. Pawlewicz, M.J., Steinshouer, D.W. & Gautier, D.L. Map showing geology, oil and gas fields, and geologic provinces of Europe including Turkey: *U.S. Geological Survey Open-File Report* **97(**470-I), 14 p. (2002).
42. Börker, J., Hartmann, J., Amman, T. & Romero-Mujalli G. Terrestrial Sediments of the Earth: Development of a Global Unconsolidated Sediments Map Database (GUM). *Geochemistry, Geophysics, Geosystems*, **19**(4), 997-1024 (2018a)
43. Börker, J., Hartmann, J., Amann, T. & Romero-Mujalli, G. Global Unconsolidated Sediments Map Database v1.0 (shapefile and gridded to 0.5° spatial resolution). *PANGAEA.* **10** (2018b)
44. Gowan, E.J., Zhang, X., Khosravi, S. *et al.* A new global ice sheet reconstruction for the past 80 000 years. *Nat Commun.* **12**, 1199 (2021).
45. Hughes, A.L.C., Gyllencreutz, R., Lohne, Ø.S., Mangerud, J., Svendsen, J.I. The last Eurasian ice sheets - a chronological database and time-slice reconstruction, DATED-1. *Boreas*, **45**(1)**,** 1-45, (2015a).
46. Hughes, A.L.C., Gyllencreutz, R., Lohne, Ø.S., Mangerud, J., Svendsen, J.I., DATED-1: compilation of dates and time-slice reconstruction of the build-up and retreat of the last Eurasian (British-Irish, Scandinavian, Svalbard-Barents-Kara Seas) Ice Sheets 40-10 ka. *Department of Earth Science, University of Bergen and Bjerknes Centre for Climate Research*, *PANGAEA*, **10** (2015b).
47. Brown, J., Ferrians, O. Heginbottom, J.A. & Melnikov, E. Circum-Arctic Map of Permafrost and Ground-Ice Conditions, Version 2. Boulder, Colorado USA. *NASA National Snow and Ice Data Center Distributed Active Archive Center*. (2002).
48. Lindgren, A., Hugelius, G., Kuhry, P., Chirstensen, T,R., Vandenberghe, J. GIS-based Maps and Area Estimates of Northern Hemisphere Permafrost Extent during the Last Glacial Maximum. *Permafrost and periglacial processes* **27** (1), 6-16. (2016).
